# Dictamnine and Related Quinoline Alkaloids from *Dictamnus*: From Phytochemistry to Biological Activities and Toxicity

**DOI:** 10.3390/molecules31213805

**Published:** 2026-10-22

**Authors:** Urszula Błaszczyk

**Affiliations:** Department of Fermentation Technology and Microbiology, Faculty of Food Technology, University of Agriculture in Krakow, Aleja Mickiewicza 21, 31-120 Krakow, Poland; urszula.blaszczyk@urk.edu.pl; Tel.: +48-12-662-47-98

**Keywords:** Dictamnine, *Cortex Dictamni*, *Dictamnus*, *Dictamnus dasycarpus*, furoquinoline alkaloids, quinoline alkaloids, phytochemicals, medicinal plants, bioactive compounds, antimicrobial activity

## Abstract

Quinoline alkaloids constitute an important and structurally diverse class of secondary metabolites occurring in the genus *Dictamnus*. Among these compounds, dictamnine is the most extensively investigated alkaloid. This review summarizes the phytochemical diversity, structural features, and biosynthetic aspects of *Dictamnus* quinoline alkaloids, with particular emphasis on the biological, pharmacological, pharmacokinetic, and toxicological properties of dictamnine. Dictamnine has been investigated for its anti-inflammatory, anticancer, antimicrobial, antifeedant, and vasorelaxant effects, involving multiple molecular and cellular mechanisms. Other quinoline alkaloids also exhibit diverse biological activities; however, their pharmacological properties and mechanisms of action remain considerably less characterized than those of dictamnine. Importantly, the biological effects of these compounds should be evaluated in the context of their safety and toxicological profiles. This review provides an overview of the current knowledge of *Dictamnus* quinoline alkaloids and highlights the need for further studies addressing their mechanisms of action, pharmacokinetic properties, and safety.

## 1. Introduction

The genus *Dictamnus* (Rutaceae) comprises aromatic perennial plants distributed predominantly across Eurasia. Among its species, *Dictamnus dasycarpus* Turcz. is particularly well known in traditional Chinese medicine. Its dried root bark, known as Bai-Xian-Pi, has long been employed for the management of various disorders, including headache, cough, edema, jaundice, rheumatic arthralgia, eczema, rubella, scabies, and other inflammatory and dermatological conditions [1,2].

Phytochemical investigations have revealed a diverse array of secondary metabolites in *Dictamnus*, including quinoline alkaloids [3,4,5,6,7,8,9,10,11,12], limonoids [3,4,7,13,14,15,16,17,18,19,20], sesquiterpenes and their glycosides [8,21,22,23,24,25,26], flavonoids [27,28,29], coumarins [3,26,27,30], steroids [3], triterpenes [16], and volatile constituents of essential oils [31,32,33,34,35], as well as other compounds [2,36]. These metabolites have been associated with a broad spectrum of biological and pharmacological effects reported for *Dictamnus*, particularly those related to root bark extracts and their individual constituents. Recent pharmacological research has focused on anti-inflammatory [37,38,39,40,41,42,43,44,45,46,47,48,49], anti-allergic [40,41,50], antimicrobial [31,32,34,51], antioxidant [32,52,53,54], anticancer [23,55,56,57,58,59], and insecticidal activities [60,61,62,63,64].

Beyond individual constituents and extracts, several studies have examined the therapeutic potential of multi-herbal combinations containing *Dictamnus dasycarpus*, reflecting its longstanding role in traditional Chinese medicine [65,66,67]. These formulations have been investigated in various pathological contexts, including atopic dermatitis, psoriasis, and bacterial infections. For example, VYAC, a multi-herbal formulation containing *D. dasycarpus*, alleviated atopic dermatitis-like symptoms by inhibiting mast cell degranulation and suppressing the Syk/NF-κB signaling pathway [65]. *D. dasycarpus* has also been included in Chinese herbal medicine baths evaluated for psoriasis vulgaris [66]. Furthermore, a three-herb medicinal extract containing *Cortex Dictamni* inhibited virulence factor production and biofilm formation by *Pseudomonas aeruginosa*, primarily by suppressing the PQS quorum-sensing system [67]. The potential chemopreventive properties of *D. dasycarpus* have also been investigated in multi-herbal formulations. Antitumor B, a six-herb combination containing *D. dasycarpus*, inhibited chemically induced oral carcinogenesis in mice, an effect associated with reduced cell proliferation and downregulation of EGFR signaling [68]. More recently, modified Zeng-Sheng-Ping (ZSP-M), which also contains *D. dasycarpus*, exhibited chemopreventive activity against oral squamous-cell carcinoma and was reported to modulate tumor-associated macrophages by targeting TNFAIP6 [69].

Among the bioactive compounds identified in the root bark of *Dictamnus*, particular attention has been devoted to the limonoids obacunone [70,71,72] and fraxinellone [57,73,74,75,76,77,78,79]. Another prominent class of secondary metabolites in *Dictamnus* comprises quinoline alkaloids, which have attracted considerable research interest owing to their diverse biological and pharmacological properties.

Against this background, the aim of the present review is to critically examine the available literature on quinoline alkaloids of the genus *Dictamnus*, with particular emphasis on dictamnine. Their phytochemical diversity, chemical structures, biosynthesis, and biological activities are reviewed, while dictamnine is further discussed in terms of its pharmacokinetics, metabolism, and toxicity. Other *Dictamnus* quinoline alkaloids are also considered to provide a broader perspective on their pharmacological potential, safety, and current research gaps.

## 2. Methodology

A literature review was conducted using databases and scientific literature platforms, including PubMed, Web of Science, ACS Publications, Google Scholar, Wiley Online Library, and Europe PMC. This review aimed to identify studies investigating the chemical, biological, pharmacological, pharmacokinetic, metabolic, and toxicological properties of dictamnine and other quinoline alkaloids reported from species of the genus *Dictamnus* L.

The literature search was performed using relevant keywords and combinations of terms related to the investigated compounds, plant taxa, and their biological and pharmacological properties. The main search terms included “dictamnine,” “Dictamni Cortex,” “Dictamnus dasycarpus,” “Dictamnus albus,” “Dictamnus angustifolius,” “furoquinoline alkaloids,” and “quinoline alkaloids.” The final literature search was conducted on 20 August 2026. Studies were included when they reported original experimental data or relevant information concerning species of the genus *Dictamnus*, *Dictamni Cortex*, dictamnine, or other quinoline alkaloids reported from *Dictamnus* species. Publications were excluded if they were unrelated to *Dictamnus* or its quinoline alkaloids, did not provide information relevant to the scope of this review, were duplicates, or concerned taxa outside the scope of this review, such as *Origanum dictamnus*. Publications for which the full text was not available in English were considered only when an English-language abstract provided sufficient information relevant to the scope of this review.

## 3. Botanical Characteristics and Phytochemistry of the Genus Dictamnus

Taxonomically, *Dictamnus* L. is classified within the family Rutaceae and comprises several Eurasian taxa. Among these, *Dictamnus dasycarpus* Turcz., *Dictamnus angustifolius* G. Don ex Sweet, and *Dictamnus albus* L. are the most extensively studied taxa, owing to their importance in traditional medicine and contemporary phytochemical and pharmacological research [1,2].

According to Plants of the World Online (POWO, Kew), *D. dasycarpus* and *D. albus* are currently accepted species, whereas *D. angustifolius* G. Don ex Sweet is treated as a synonym of *D. albus* [80]. Throughout this review, taxon names are retained as used in the original source publications to ensure consistency with the published data and accurate attribution of the findings.

*D. dasycarpus* is an East Asian species whose natural distribution encompasses northeastern China, the Korean Peninsula, Mongolia, and parts of southeastern Siberia [1,2]. In contrast, *D. albus* L. is the most widely distributed representative of the genus in Europe, occurring from Central and Southern Europe through parts of Western Asia to southwestern Siberia and the western Himalayas [81]. Other taxa associated with the genus *Dictamnus* have also been described in the literature, including *Dictamnus caucasicus* (Boiss.) Fisch. ex Grossh., distributed in the Caucasus and Iran, and *Dictamnus hispanicus* Webb ex Willk., occurring primarily in the Iberian Peninsula [1,81]. According to the current taxonomic treatment adopted by POWO, *D. caucasicus* is treated as a synonym of *D. albus*, whereas *D. hispanicus* is recognized as an accepted species [80].

Plants of the genus *Dictamnus* are characterized by a diverse phytochemical profile comprising numerous classes of secondary metabolites. To date, more than 200 natural compounds have been identified, predominantly from *D. dasycarpus*, including alkaloids, limonoids, sesquiterpenes, coumarins, flavonoids, triterpenoids, steroids, and volatile constituents of essential oils [3,5,9,12]. The composition and relative abundance of secondary metabolites may vary substantially among *Dictamnus* species. Lv et al. [82] demonstrated marked differences in the chemical profiles of the root bark of *D. dasycarpus* and *D. angustifolius*, with *D. angustifolius* containing higher levels of several major furoquinoline alkaloids. Moreover, extracts of *D. angustifolius* showed stronger cytotoxic, antimicrobial, and antioxidant activities, suggesting that differences in furoquinoline alkaloid composition may contribute to the variation in biological activity observed between these species.

A distinctive feature of *Dictamnus* is the production and release of volatile terpenoids. The aerial parts contain secretory cavities [83] and are covered with glandular trichomes involved in the biosynthesis, storage, and secretion of essential oils [84]. The abundance of volatile terpenoids facilitates their release, particularly under high-temperature conditions. The flammability of the volatile compounds released by these plants is reflected in the common English names “burning bush” and “gas plant” associated with *Dictamnus albus* [85]. The essential oils of *Dictamnus* species consist predominantly of monoterpenes and sesquiterpenes, with their qualitative and quantitative profiles varying according to species, geographical origin, and plant part analyzed [31,32,33,34,35].

## 4. Quinoline Alkaloids of Dictamnus

### 4.1. Chemical Structure and Structural Diversity of Quinoline Alkaloids from Dictamnus

**Figure 1:**
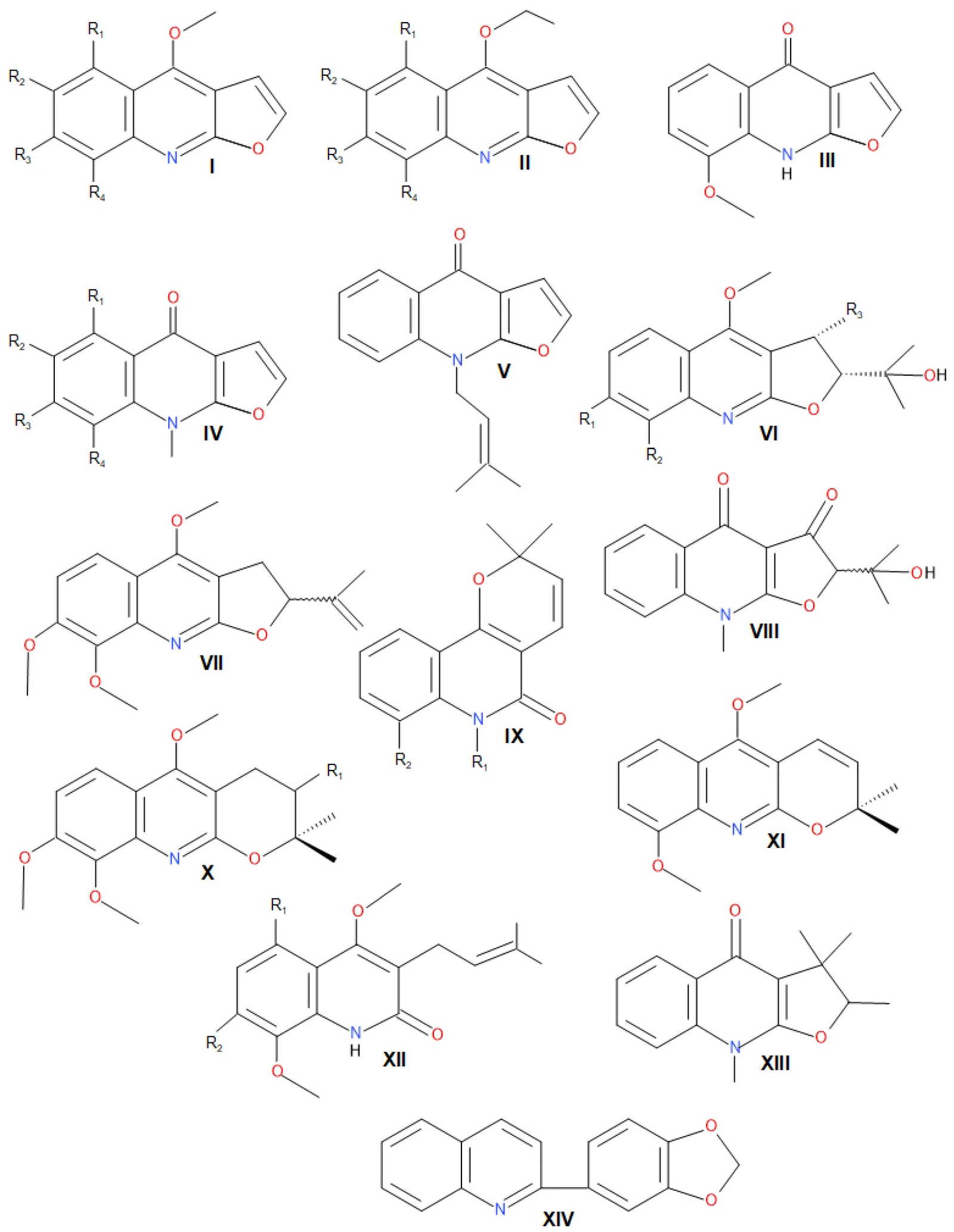
Structure of main quinoline alkaloids from *Dictamnus*.

**Figure 2:**
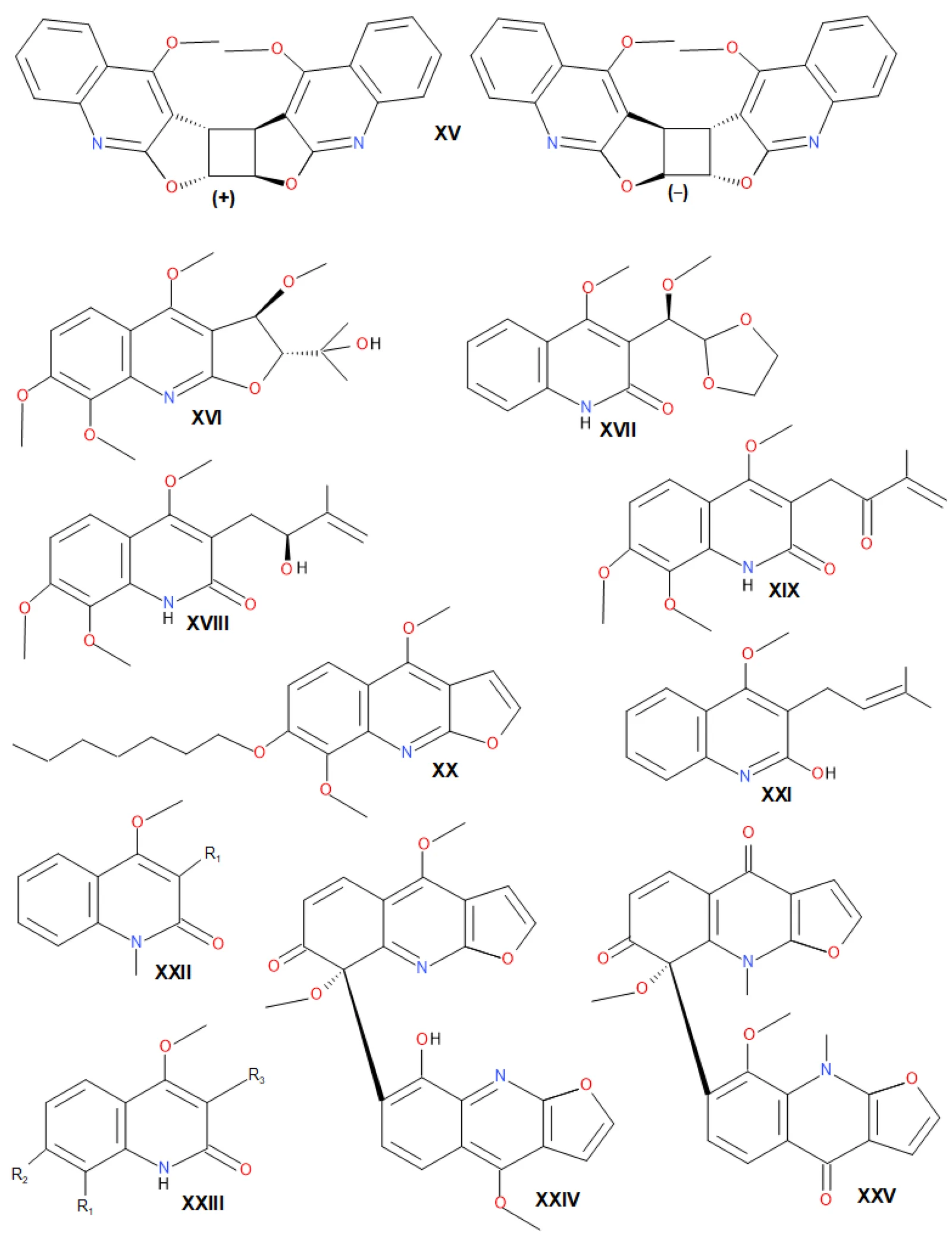
Structure of main quinoline alkaloids from *Dictamnus* (continued).

Among the diverse secondary metabolites isolated from representatives of the genus *Dictamnus*, quinoline alkaloids represent a particularly important class of compounds. The quinoline alkaloids identified in *Dictamnus* species, together with the corresponding species in which they occur, are summarized in Table 1, while representative chemical structures are presented in Figure 1 and Figure 2.

Based on their structural characteristics, the quinoline alkaloids identified in *Dictamnus* can be broadly classified into simple quinoline, furoquinoline, quinolone (quinolinone), and pyran-containing quinoline alkaloids. Furoquinoline alkaloids are characterized by a fused quinoline–furan ring system. They constitute the predominant group of alkaloids reported from *Dictamnus* species and are also characteristic of other representatives of the family Rutaceae [105]. Quinoline alkaloids identified in the genus *Dictamnus* also include pyran-containing derivatives, such as pyranoquinolinone and pyranoquinoline derivatives.

The structural diversity of quinoline alkaloids in *Dictamnus* is primarily determined by differences in the number, position, and nature of substituents present within the quinoline and furoquinoline scaffolds. Methoxy (-OCH_3_), hydroxy (-OH), and methyl (-CH_3_) groups are particularly common and contribute to differences in the polarity, lipophilicity, and other physicochemical properties of individual compounds. Overall, variations in substitution patterns, the presence of oxygen-containing substituents, and the mode of ring fusion give rise to a broad range of structurally related compounds.

Methoxy substitution is one of the most common structural modifications among quinoline alkaloids in *Dictamnus*. The position of methoxy groups may influence the physicochemical properties and biological activities of individual compounds. For example, Nam et al. [92] demonstrated that differences in the position of methoxy groups may affect the inhibition of phosphodiesterase 5A (PDE5A) by furoquinoline alkaloids. In another study investigating the mutagenicity of furoquinoline alkaloids, a decrease in mutagenic activity was observed with an increasing number of methoxy substituents on the furoquinoline skeleton, as demonstrated for dictamnine, 7-fagarine, and skimmianine [106]. Together, these observations suggest that relatively subtle structural modifications may influence the biological properties of closely related alkaloids.

The fused furan ring is a characteristic structural feature of furoquinoline alkaloids that distinguishes them from simpler quinoline and quinolone derivatives. Importantly, the presence of a furan ring may significantly influence the metabolism of these alkaloids. Oxidation of the furan ring by cytochrome P450 enzymes may lead to the formation of reactive intermediates and may contribute to the potential toxicity of these compounds [107].

Additional structural diversity is provided by glycosylated quinoline derivatives, such as 3-[1β-hydroxy-2-(β-D-glucopyranosyloxy)ethyl]-4-methoxy-2(1H)-quinolinone [95]. The attachment of a sugar residue represents a distinct structural modification that increases molecular polarity and may alter solubility and other physicochemical properties. Further structural complexity is observed in dimeric furoquinoline alkaloids. Sun et al. [10] isolated anstifolines A and B from the root bark of *D. angustifolius* (Figure 2, structures XXIV and XXV). Another quinoline alkaloid, dasycarine A, isolated from *D. dasycarpus* (Figure 2, structure XV), represents a rare type of dimeric furoquinoline alkaloid formed through [2 + 2] cycloaddition involving the furan ring, resulting in the dimerization of dictamnine [5]. These compounds contain two furoquinoline-derived units linked through unusual coupling patterns, highlighting the remarkable structural diversity of quinoline alkaloids in *Dictamnus* and demonstrating that their structural diversification extends beyond modifications in individual monomeric alkaloids.

Among the quinoline alkaloids reported from *Dictamnus*, dictamnine, γ-fagarine, skimmianine, robustine, preskimmianine, haplopine, isomaculosidine, and isodictamnine are among the most frequently isolated compounds (Table 1). Their occurrence and relative abundance may vary depending on the species, plant organ, developmental stage, and extraction conditions.

**Figure 3:**
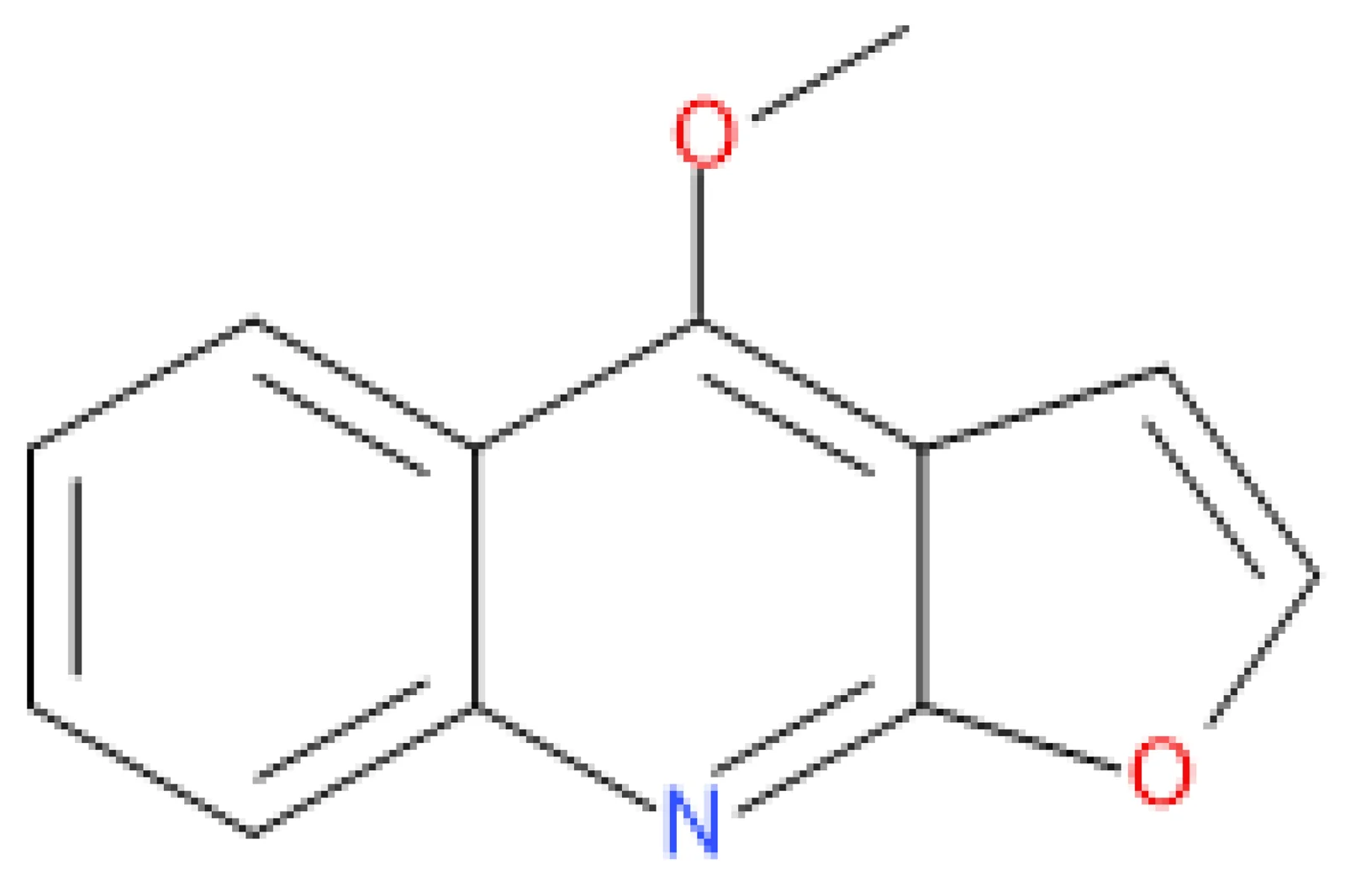
Structure of dictamnine.

Dictamnine (4-methoxyfuro [2,3-b]quinoline) is a furoquinoline alkaloid (Figure 3) characteristic of species of the genus *Dictamnus*. This compound has also been reported from other genera of the family Rutaceae, including *Ruta*, *Haplophyllum*, *Zanthoxylum*, *Esenbeckia*, and *Euodia* [108,109,110,111,112,113]. Its molecule contains a fused furo [2,3-*b*]quinoline ring system and a methoxy group at C-4, with a molecular weight of 199.20 g/mol. Dictamnine was first isolated from the root of *Dictamnus albus* L. [114]. It was subsequently detected and isolated from other species of the genus [3,4,86,87,88,89].

### 4.2. Extraction and Isolation of Quinoline Alkaloids from Dictamnus

The isolation of quinoline alkaloids from *Dictamnus* species has generally involved solvent extraction of roots or root bark, often followed by liquid–liquid partitioning and subsequent chromatographic fractionation and purification [3,4,5,6,7,8,9,10,11,12]. Ethanol or aqueous ethanol was commonly used for the initial extraction. Depending on the study, the crude extracts were subjected to liquid–liquid partitioning between aqueous and organic phases using solvents of varying polarity, such as petroleum ether, dichloromethane, ethyl acetate, and n-butanol, or were directly subjected to chromatographic separation. The obtained fractions were further separated or purified using, among other techniques, silica gel column chromatography, Sephadex LH-20, reversed-phase chromatography on ODS (C18) columns, preparative thin-layer chromatography, and, in some cases, semi-preparative HPLC [3,4,5,6,7,8,9,10,11,12]. Crystallization or recrystallization was also occasionally employed for purification [5,6,12,91,104].

Alternative extraction and separation approaches have also been investigated. Jiang et al. [87] developed pressurized liquid extraction (PLE) followed by HPLC for the simultaneous determination of seven active constituents in *Cortex Dictamni* [87]. In contrast, Wang et al. [88] combined supercritical fluid extraction (SFE) of *D. dasycarpus* root bark with high-speed counter-current chromatography (HSCCC), enabling the isolation of dictamnine and other constituents [88].

The isolated compounds were generally identified and their structures characterized using spectroscopic and spectrometric techniques, including NMR, mass spectrometry, IR, and UV spectroscopy, with additional methods applied where appropriate, such as comparison with previously reported data, ECD analysis, and single-crystal X-ray diffraction [3,4,5,6,7,8,9,10,11,88,91,104].

### 4.3. Biosynthesis of Quinoline Alkaloids in Dictamnus

The biosynthesis of quinoline alkaloids in *Dictamnus* remains incompletely characterized. Recent transcriptomic and metabolomic analyses of *Dictamnus dasycarpus* have provided insights into metabolic pathways associated with quinoline alkaloid accumulation and identified candidate genes potentially involved in these pathways [115].

From a general biosynthetic perspective, quinoline alkaloids have been associated with aromatic precursors derived from tryptophan metabolism, particularly anthranilate-related intermediates [116]. In *D. dasycarpus*, Mao et al. [115] identified differentially expressed genes associated with tryptophan biosynthesis and metabolism and proposed 4-methoxy-2(1H)-quinolinone as a potential intermediate in quinoline alkaloid biosynthesis. Candidate genes, including HAC1, NAGS1, and NAGS2, showed expression patterns associated with the accumulation of this quinolinone. However, these observations indicate metabolic associations rather than experimentally validated enzymatic steps.

Supporting biochemical evidence for anthranilate-derived quinolone formation comes from studies in other plant species. Abe et al. [117] demonstrated that benzalacetone synthase from *Rheum palmatum*, a type III polyketide synthase, catalyzes the condensation of *N*-methylanthraniloyl-CoA or anthraniloyl-CoA with malonyl-CoA or methylmalonyl-CoA to produce 4-hydroxy-2(1H)-quinolone derivatives. Although this reaction has not been experimentally demonstrated in *D. dasycarpus*, it provides biochemical support for the possible involvement of anthranilate-derived CoA thioesters in the formation of quinolone-type scaffolds. Further supporting evidence comes from the Rutaceae family. Junghanns et al. [118] isolated and cloned a cDNA encoding acridone synthase from cell suspension cultures of *Ruta graveolens* and confirmed its identity by heterologous expression in *Escherichia coli*. The recombinant enzyme catalyzed the formation of 1,3-dihydroxy-N-methylacridone from N-methylanthraniloyl-CoA and malonyl-CoA. This finding provides biochemical evidence that anthranilate-derived CoA thioesters can serve as precursors for the formation of nitrogen-containing aromatic scaffolds in Rutaceae. However, because the reaction was demonstrated in *R. graveolens* and involves an acridone instead of a quinolinone scaffold, it should be regarded as supporting rather than direct evidence for quinoline alkaloid biosynthesis in *Dictamnus*.

Following the formation of the quinolinone-type scaffold, subsequent tailoring reactions are likely to contribute substantially to the structural diversity of quinoline alkaloids. In *D. dasycarpus*, Mao et al. [115] identified candidate methyltransferases, isomerases, and cytochrome P450 monooxygenases (CYP450s) whose expression was associated with the accumulation of quinoline alkaloids. These enzymes may participate in oxidation, isomerization, acetylation, and other tailoring reactions involved in the structural diversification of alkaloids. However, the specific enzyme–substrate relationships have not yet been experimentally established in *D. dasycarpus*.

The biosynthesis of furoquinoline alkaloids remains poorly characterized. These alkaloids contain a fused quinoline–furan ring system, and their formation has been proposed to involve the incorporation of an isoprenoid-derived unit into a quinoline precursor, followed by cyclization to form the furan ring [105].

Evidence supporting this proposed sequence comes primarily from biosynthetic studies in other plant species. Earlier biosynthetic studies provided experimental support for a more specific mechanism of furan-ring formation. Grundon et al. [119] suggested that the furan ring in furoquinoline alkaloids arises through oxidative cyclization of a prenyl derivative to a hydroxyisopropyldihydrofuran, followed by loss of the isopropyl fragment. Their isotope-labeling experiments further provided evidence for the role of dictamnine as a biosynthetic intermediate in the formation of skimmianine in *Choisya ternata*. The authors also proposed that aromatic hydroxylation in the pathway to oxygenated furoquinoline alkaloids may occur after formation of the furan ring [120]. A proposed pathway therefore involves prenylation of a hydroxyquinoline intermediate, followed by oxidative cyclization and subsequent rearrangement and loss of the isopropyl-derived fragment to generate the fused furan ring. However, although the isotope-labeling experiments of Grundon et al. [119] provide important experimental evidence for precursor–product relationships in furoquinoline alkaloid biosynthesis, the enzymatic mechanisms underlying these transformations have not been established in *Dictamnus*. Similarly, the specific prenyltransferases, oxidases, cyclases, and other enzymes responsible for these reactions remain unidentified in *D. dasycarpus*. Thus, the proposed pathway provides a useful framework for interpreting the structural diversity of furoquinoline alkaloids such as dictamnine, skimmianine, and γ-fagarine, but its individual enzymatic steps remain to be experimentally validated in this genus [105,115,119].

## 5. Biological Activities of Dictamnine

Dictamnine exhibits a broad spectrum of biological activities, including anti-inflammatory, immunomodulatory, anticancer, antimicrobial, antifungal, and vasorelaxant effects. The available evidence suggests that these activities are mediated through multiple molecular and cellular mechanisms, although the strength of evidence varies considerably among biological effects and experimental models. An overview of the biological activities of dictamnine, including the experimental models, mechanisms of action, and main findings reported in the literature, is presented in Table 2.

### 5.1. Anti-Inflammatory and Immunomodulatory Activities of Dictamnine

Persistent or excessive inflammation contributes to the pathogenesis of numerous diseases, including atopic dermatitis (AD), psoriasis, asthma, inflammatory bowel diseases, rheumatoid arthritis (RA), osteoarthritis (OA), and other immune-mediated disorders. Increasing evidence suggests that dictamnine may exert anti-inflammatory and immunomodulatory effects by modulating signaling pathways involved in inflammation, immune-cell function, oxidative stress, regulated cell death, and neuroimmune mechanisms.

Atopic dermatitis (AD) is a multifactorial chronic inflammatory skin disease in which immune dysregulation, epidermal barrier dysfunction, oxidative stress, and neuroimmune interactions contribute to disease initiation, progression, and chronicity [139]. One of the best-characterized mechanisms underlying the anti-inflammatory activity of dictamnine in AD involves the modulation of macrophage function. Macrophages exhibit considerable functional plasticity, and their phenotype and activity are shaped by the local microenvironment and multiple stimuli.

Huang et al. [120] demonstrated that dictamnine reduced M1 macrophage polarization and enhanced autophagy. In a dinitrofluorobenzene (DNFB)-induced model of AD, these effects were associated with reduced inflammatory cell infiltration, decreased levels of pro-inflammatory mediators, and improvement in histopathological skin changes. In LPS/IFN-γ-stimulated M1 macrophages, dictamnine increased the expression of microtubule-associated protein 1A/1B light chain 3 (LC3) and reduced both the production of IL-1β, TNF-α, IL-6, MCP-1, and IL-8. These findings suggest that modulation of macrophage function and autophagy contributes to the anti-inflammatory effects of dictamnine in experimental AD [120].

Another important aspect of dictamnine activity in AD is its potential effect on chronic pruritus, one of the most burdensome symptoms of the disease. Yang et al. [123] demonstrated that dictamnine alleviated pruritus and reduced epidermal thickening and inflammatory cell infiltration in an experimental AD model. These effects were associated with reduced expression of MrgprA3 and TRPA1 and decreased excitability of neurons expressing these receptors. MrgprA3-expressing sensory neurons are an important component of histamine-independent itch signaling, while TRPA1 acts as a key ion channel functioning at downstream stages of this signaling pathway. Molecular docking analysis indicated a potential interaction between dictamnine and the MrgprA3 receptor, supporting the hypothesis that dictamnine may modulate the MrgprA3–TRPA1 pathway [123]. However, this docking-based prediction should be interpreted with caution, as molecular docking provides evidence of a potential molecular interaction but does not establish direct receptor binding or pharmacological affinity [123].

The anti-inflammatory activity of dictamnine in AD is further supported by comparative studies with fraxinellone. Chou et al. [121] evaluated both compounds in cellular models and in an oxazolone-induced mouse model of AD. Both compounds exerted anti-inflammatory effects and reduced the severity of skin lesions, including erythema, edema, inflammatory cell infiltration, and pruritus, although their pharmacological profiles differed. Dictamnine had a more pronounced effect on inflammatory responses in keratinocytes and macrophages, whereas fraxinellone more strongly inhibited basophil degranulation and allergic responses. These findings suggest that dictamnine may act on several cellular components involved in cutaneous inflammation, while fraxinellone may have a more prominent effect on pathways associated with allergic responses [121].

The efficacy of dictamnine in inflammatory skin disorders may also depend on its mode of delivery. Lin et al. [122] demonstrated that poly(lactic-co-glycolic acid) (PLGA)-based nanocarriers enhanced the local delivery and skin penetration of dictamnine and improved its efficacy in a model of contact dermatitis. Controlled release at the site of inflammation was associated with attenuation of the inflammatory response, including reduced levels of selected pro-inflammatory cytokines and decreased activity of pathways associated with NLRP3 inflammasome and NF-κB signaling. These findings suggest that, in addition to the intrinsic pharmacological activity of dictamnine, its local bioavailability and delivery strategy may influence its therapeutic efficacy [122].

Recent evidence has further expanded the potential anti-inflammatory and anti-psoriatic profile of dictamnine. Wang et al. [140] investigated the transdermally permeable constituents of *Dictamni Cortex* and identified 26 such compounds among 41 characterized constituents. Network pharmacology analysis identified potential therapeutic targets for psoriasis, including TNF, MMP9, ICAM1, TLR4, EGFR, and MAPK14, while molecular docking was used to select 6 transdermal constituents with potential anti-psoriatic activity, including dictamnine [140]. In vitro assays further showed that these compounds suppressed abnormal cell proliferation and reduced inflammatory factor levels in a psoriasis-like HaCaT cell model. Based on a comparative evaluation of multiple indicators, dictamnine was identified by the authors as the compound with the most promising anti-psoriatic activity [140].

Further evidence from Wang et al. [127] supports the anti-psoriatic activity of dictamnine. In a mouse model of imiquimod-induced psoriasis-like dermatitis, dictamnine significantly alleviated skin lesions. The observed effects were associated with modulation of HIF1A, suppression of HIF1A nuclear translocation, and reduced expression of the ferroptosis-related proteins GPX4 and SLC7A11. The effects of dictamnine were attenuated by the ferroptosis inhibitor ferrostatin-1 and the HIF1A stabilizer DMOG, providing experimental support for the involvement of HIF1A-dependent ferroptosis in its anti-psoriatic activity [127]. These findings suggest that dictamnine may exert anti-psoriatic effects by suppressing keratinocyte hyperproliferation and inflammatory mediator production, at least in part through modulation of HIF1A-dependent ferroptosis. However, the precise molecular targets underlying these effects require further experimental validation [127].

In contrast, in DSS-induced experimental colitis, dictamnine activated the Nrf2/GPX4 pathway, thereby limiting ferroptosis in intestinal epithelial cells and reducing pathological tissue damage [126]. Dictamnine also improved intestinal barrier-related parameters, reduced the production of inflammatory cytokines, and enhanced antioxidant responses. These effects were accompanied by increased GPX4 expression and activation of Nrf2. In vitro experiments further supported a protective effect of dictamnine against ferroptotic injury in intestinal epithelial cells. Thus, inhibition of ferroptosis through activation of the Nrf2/GPX4 axis may contribute to the protective effects of dictamnine in experimental ulcerative colitis [126].

Interestingly, the effects of dictamnine on ferroptosis appear to differ across cell types, tissues, and disease contexts. In experimental colitis, inhibition of ferroptosis was associated with protection of intestinal epithelial cells, whereas in the psoriasis model, induction of ferroptosis contributed to the inhibition of keratinocyte hyperproliferation. Experimental conditions also differed between the models, including the disease-inducing stimuli and cell systems: DSS-induced colitis and RSL3-induced ferroptotic injury in NCM460 cells were used in the colitis study, whereas imiquimod-induced psoriasis-like dermatitis and KGF-stimulated HaCaT keratinocytes were used in the psoriasis study. These differences may contribute to the divergent effects of dictamnine on ferroptosis, although the relative contribution of cell type, tissue microenvironment, disease-associated signaling, and experimental conditions remains unclear.

The role of ferroptosis in the biological activity of dictamnine has also been investigated in osteoarthritis. Guo et al. [129] demonstrated that dictamnine alleviated osteoarthritis progression by inhibiting chondrocyte ferroptosis. In vitro, treatment with 50 or 100 μM dictamnine reduced ferroptotic changes in chondrocytes, while its protective effects were further supported in an animal model of OA and validated using human samples. Mechanistically, the chondroprotective effects of dictamnine were associated with modulation of the HIF-1α/HO-1 signaling pathway. These findings suggest that inhibition of chondrocyte ferroptosis may contribute to the anti-osteoarthritic activity of dictamnine. However, this mechanism should be distinguished from direct anti-inflammatory effects, as the primary outcomes of the study concerned ferroptosis and OA progression rather than inflammation itself.

Beyond the regulation of ferroptosis, dictamnine has also been investigated in other inflammatory conditions, including allergic asthma. Jung et al. [124] demonstrated that dictamnine affected the TGF-β/Smad2/3 signaling pathway, which is involved in airway remodeling, fibrosis, and persistent inflammation. Oral administration of dictamnine reduced airway hyperresponsiveness, inflammatory cell infiltration, mucus production, and peribronchial fibrosis in an ovalbumin-induced mouse model. These effects were accompanied by reduced TGF-β and phosphorylated Smad2/3 expression and increased ZO-1 expression. In human bronchial epithelial cells, dictamnine also reduced TGF-β-induced Smad2/3 phosphorylation. Network pharmacology further identified the TGF-β signaling pathway among the pathways potentially associated with dictamnine [124]. These findings suggest that dictamnine may influence both inflammatory responses and airway remodeling, although the available evidence does not establish a direct effect on allergic sensitization.

A related anti-allergic effect has also been reported in allergic rhinitis. Liu et al. [125] demonstrated that dictamnine inhibited IgE/FcεRI-induced mast cell activation in LAD2 cells and attenuated allergic responses in an ovalbumin (OVA)-induced murine model of allergic rhinitis. At the molecular level, dictamnine reduced the activation of the tyrosine kinase LYN and downstream phosphorylation of PLCγ1, PKC, IP3R, ERK1/2, and Akt. In vivo, dictamnine reduced nasal rubbing and sneezing and attenuated OVA-induced allergic responses. These findings suggest that dictamnine may exert anti-allergic effects, at least in part, by suppressing FcεRI–LYN-mediated mast cell signaling.

Another example of the anti-inflammatory and immunomodulatory activity of dictamnine is provided by studies of rheumatoid arthritis. Wang et al. [128] evaluated dictamnine in vitro using human rheumatoid arthritis fibroblast-like synoviocytes (HFLS-RA) stimulated with TNF-α and in vivo using a collagen-induced arthritis (CIA) mouse model. Dictamnine inhibited TNF-α-induced proliferation of HFLS-RA cells and reduced pathological synovial hyperplasia in the CIA model. Proteomic and mechanistic analyses implicated the nuclear receptor NR1D1 (Rev-erbα) in these effects and suggested its involvement in the regulation of oxidative stress. Modulation of the NR1D1/Keap1/Nrf2/ARE axis was associated with increased expression of antioxidant genes, including HO-1, reduced intracellular ROS levels, and improved mitochondrial function. Pharmacological inhibition and siRNA-mediated silencing of NR1D1 attenuated or abolished the protective effects of dictamnine, supporting an important role for NR1D1 in the proposed mechanism [128]. These findings indicate that dictamnine may exert anti-inflammatory and antiproliferative effects in experimental RA by limiting synovial hyperplasia through reduction in oxidative stress and modulation of the NR1D1/Keap1/Nrf2/ARE axis. Nevertheless, the relevance of this mechanism to human RA remains to be established.

### 5.2. Anticancer Activity of Dictamnine

Cancer remains one of the leading causes of mortality worldwide, highlighting the need for novel anticancer compounds with high efficacy and acceptable safety profiles. Increasing evidence suggests that dictamnine may exert multifaceted anticancer effects by involving modulation of tumor-cell-intrinsic signaling pathways and components of the tumor microenvironment. Preclinical studies have reported its activity in lung, pancreatic, colorectal, and prostate cancer models, with proposed mechanisms including inhibition of cancer cell proliferation, induction of apoptosis and ferroptosis, suppression of epithelial–mesenchymal transition (EMT), inhibition of migration and invasion, and modulation of tumor-associated macrophages. However, the available evidence remains predominantly preclinical, and the clinical relevance of these findings has yet to be established.

One of the best-characterized aspects of the anticancer activity of dictamnine is its antiproliferative effect. Aberrant activation of the PI3K/AKT/mTOR and MAPK signaling pathways is frequently observed in cancer and contributes to uncontrolled proliferation and tumor progression. Experimental evidence suggests that dictamnine can modulate these pathways in several cancer models.

Yu et al. [130] identified dictamnine as an inhibitor of the c-Met receptor, a receptor tyrosine kinase implicated in the development and progression of numerous epithelial malignancies. Inhibition of c-Met signaling was associated with reduced phosphorylation of components of the PI3K/AKT/mTOR pathway and decreased activity of ERK and p38, downstream components of MAPK signaling. These molecular changes were accompanied by reduced proliferation of lung cancer cells and inhibition of tumor growth in both cellular and animal models. Thus, the antiproliferative activity of dictamnine in lung cancer appears to be associated with modulation of multiple signaling pathways downstream of c-Met.

Similar effects have been reported in pancreatic cancer. Zhang et al. [134] demonstrated that dictamnine inhibited PI3K/AKT signaling, resulting in reduced proliferation, migration, and invasion of pancreatic cancer cells. Taken together, these findings suggest that modulation of PI3K/AKT/mTOR and MAPK signaling may contribute to the antiproliferative activity of dictamnine across different tumor types. Nevertheless, further studies are required to determine whether these effects reflect a common molecular mechanism or cancer-type-specific responses.

Induction of apoptosis represents another important mechanism underlying the anticancer activity of dictamnine. Wang et al. [131] reported that dictamnine increased apoptosis in lung cancer cells, accompanied by modulation of Bcl-2 family proteins and activation of executioner caspases. The study also demonstrated reduced expression of hypoxia-inducible factor 1-alpha (HIF-1α), a transcription factor involved in tumor adaptation to hypoxia, angiogenesis, and tumor progression. Consistent with these observations, Zhang et al. [134] reported activation of caspase-3 and an increased Bax/Bcl-2 ratio following dictamnine treatment in pancreatic cancer cells.

The epithelial–mesenchymal transition (EMT) is a key process contributing to tumor invasion and metastasis. During EMT, epithelial cancer cells lose cell–cell adhesion and acquire mesenchymal characteristics that facilitate migration and invasion. Several studies suggest that dictamnine interferes with this phenotypic transition. Wang et al. [131] demonstrated that dictamnine increased E-cadherin expression while decreasing N-cadherin, vimentin, and Slug expression, accompanied by reduced migration and invasiveness of lung cancer cells. Similar findings were reported in pancreatic cancer by Zhang et al. [134], in which dictamnine increased E-cadherin expression and reduced N-cadherin, vimentin, and Snail expression, together with inhibition of migration and invasion. More recently, He et al. [135] found that dictamnine suppressed EMT progression in prostate cancer, as evidenced by increased E-cadherin and decreased vimentin expression. These effects were associated with inhibition of Wnt/β-catenin signaling through stabilization of DKK1 and were accompanied by reduced migration and invasion of prostate cancer cells. These findings support the hypothesis that suppression of EMT contributes to the anti-invasive effects of dictamnine. However, further studies are needed to determine whether EMT inhibition represents a primary effect of dictamnine or a downstream consequence of broader alterations in cellular signaling.

The anticancer activity of dictamnine may also involve the tumor microenvironment, particularly tumor-associated macrophages (TAMs), which play important roles in tumor progression, angiogenesis, immune suppression, and metastasis. Zuo et al. [132] demonstrated that dictamnine inhibited macrophage polarization toward the M2 phenotype in a colorectal cancer model while simultaneously promoting ferroptosis in colorectal cancer cells. These activities were associated with modulation of MAPK signaling and inhibition of tumor growth, suggesting that dictamnine may exert both direct effects on tumor cells and immunomodulatory effects within the tumor microenvironment. However, evidence regarding these immunomodulatory properties remains limited compared with that concerning direct effects on cancer cells.

Ferroptosis represents another mechanism through which dictamnine may induce cancer cell death. This iron-dependent form of regulated cell death is characterized by oxidative stress and excessive lipid peroxidation and is mechanistically distinct from apoptosis. In the study by Zuo et al. [132], dictamnine increased reactive oxygen species levels and promoted lipid peroxidation in colorectal cancer cells, resulting in ferroptotic cell death. These findings suggest that ferroptosis may represent an additional mechanism contributing to the anticancer activity of dictamnine alongside apoptosis. However, evidence for dictamnine-induced ferroptosis remains limited, and further studies are required to identify the molecular targets involved and determine the contribution of ferroptosis to the overall antitumor effect, particularly in vivo.

The potential of dictamnine to enhance the efficacy of other anticancer compounds has also been investigated. An et al. [133] reported that combined treatment with dictamnine and dihydroartemisinin markedly increased apoptotic cell death in human A549 lung adenocarcinoma cells compared with dictamnine treatment alone. This enhanced apoptotic response was associated with increased caspase-3 activation, whereas inhibition of caspase-3 significantly attenuated combination-induced apoptosis, suggesting that dihydroartemisinin may potentiate dictamnine-induced apoptosis through a caspase-dependent mechanism.

### 5.3. Antibacterial and Antifungal Activity of Dictamnine

The increasing resistance of microorganisms to commonly used antimicrobial agents represents a major challenge for modern medicine, highlighting the need for new antimicrobial strategies. Dictamnine has demonstrated antibacterial and antifungal activity against several microorganisms and may also attenuate pathogen virulence [136,137]. In contrast to conventional antimicrobial agents that primarily target essential cellular processes, dictamnine may interfere with mechanisms involved in pathogen colonization and persistence. In particular, its activity against uropathogenic *Escherichia coli* (UPEC) appears to involve inhibition of bacterial adhesion and invasion rather than direct bactericidal activity. The proposed anti-virulence effect may attenuate pathogen virulence without directly affecting bacterial viability; however, whether dictamnine can reduce the emergence of antimicrobial resistance remains to be established.

The best-characterized activity of dictamnine against UPEC involves inhibition of bacterial adhesion and invasion. The pathogenesis of UPEC infection begins with adhesion to urinary tract epithelial cells, followed by invasion of urothelial cells. Intracellular bacterial communities and reservoirs formed during this process can contribute to persistence and recurrence of infection. Yang et al. [136] demonstrated that dictamnine reduced both adhesion and invasion of UPEC strains into bladder epithelial cells. Importantly, these effects were observed at concentrations that did not cause substantial cytotoxicity to host cells, suggesting a degree of selectivity for pathogen-associated processes.

The mechanism underlying the activity of dictamnine against UPEC appears to differ from that of conventional bactericidal antibiotics. Yang et al. [136] showed that dictamnine did not primarily act by directly eliminating bacteria but instead impaired bacterial colonization by interfering with interactions between bacterial adhesins and receptors on epithelial cells. Reduced bacterial adhesion was accompanied by a decrease in bacterial invasion of host cells and, consequently, reduced establishment of infection. These findings support the classification of dictamnine as a potential anti-virulence compound, although further studies are required to determine its precise molecular targets and to establish whether this mechanism is maintained in vivo.

Inhibition of UPEC adhesion and invasion may be particularly relevant to recurrent urinary tract infections, as these processes contribute to the establishment of intracellular bacterial reservoirs. However, whether dictamnine can prevent reservoir formation or reduce recurrent infection remains to be demonstrated in appropriate in vivo models.

Beyond UPEC, dictamnine has also demonstrated inhibitory activity against several other microorganisms. Tian et al. [6] reported moderate activity against *Bacillus subtilis* (ATCC 6633), *Candida albicans* (ATCC 10231), and *Pseudomonas aeruginosa* (ATCC 27853), with minimum inhibitory concentrations (MICs) of 32, 64, and 64 µg/mL, respectively. These findings indicate that dictamnine possesses a broader spectrum of antimicrobial activity; however, the relatively moderate MIC values and the absence of detailed mechanistic data limit conclusions regarding its therapeutic applicability. They are consistent with the reported antimicrobial properties of *Dictamnus dasycarpus* preparations, although they do not provide evidence of clinical efficacy.

Antifungal activity of dictamnine has also been reported against phytopathogenic fungi. Zhao et al. [20] found that dictamnine exhibited strong antifungal activity against *Cladosporium cucumerinum* and was the most active compound among the metabolites isolated from *Dictamnus dasycarpus*, with activity observed at an amount of 0.6 µg per TLC plate. Although this study provides evidence of antifungal activity, the experimental conditions and assay format differ from contemporary standardized susceptibility testing, making direct comparison with MIC values from more recent studies difficult.

More recently, Liu et al. [137] demonstrated substantial activity of dictamnine against *Botrytis cinerea*, a phytopathogenic fungus responsible for gray mold and significant economic losses during fruit storage. Although this study addressed plant pathology rather than human fungal infection, it provides evidence for the antifungal activity of dictamnine and offers insights into its possible mechanism of action.

Liu et al. [137] associated the antifungal activity of dictamnine with disruption of ergosterol biosynthesis. Ergosterol is the major sterol of the fungal cell membrane and is essential for maintaining membrane integrity, fluidity, and normal cellular function. Impairment of ergosterol biosynthesis can lead to increased membrane permeability, disruption of cellular homeostasis, and inhibition of fungal growth. Gene expression analyses indicated downregulation of genes encoding enzymes involved in the ergosterol biosynthetic pathway, which was accompanied by reduced ergosterol content and inhibition of fungal development [137].

Dictamnine exposure was also associated with alterations in fungal membrane organization and increased membrane permeability. These changes resulted in leakage of intracellular components and inhibition of hyphal growth, ultimately reducing fungal development on stored fruit. The authors therefore proposed that dictamnine may have potential as a natural plant-protection compound and could potentially contribute to strategies aimed at reducing the use of synthetic fungicides. However, these findings should not be directly extrapolated to the treatment of human fungal infections. The proposed antifungal mechanism shares certain features with that of azole antifungals, which also interfere with ergosterol biosynthesis. However, the precise molecular target of dictamnine remains unclear, and it has not been established whether dictamnine directly inhibits enzymes involved in sterol biosynthesis or instead affects their expression or regulation.

### 5.4. Vasorelaxant Activity of Dictamnine

Dysregulation of vascular tone contributes to the pathogenesis of numerous cardiovascular diseases. In addition to its anti-inflammatory, antimicrobial, and anticancer activities, dictamnine has been reported to exert vasorelaxant effects in experimental vascular models. Yu et al. [138] demonstrated that dictamnine induced concentration-dependent relaxation of isolated rat aortic rings. The vasorelaxant effect was associated with inhibition of calcium influx into vascular smooth muscle cells, suggesting that modulation of calcium-dependent contractile mechanisms contributes to the observed response. Comparison with cromakalim and calcium channel antagonists further suggested a possible involvement of potassium channels; however, the precise molecular mechanism underlying the vasorelaxant activity of dictamnine remains unclear. These findings suggest that dictamnine may modulate vascular tone through calcium-dependent mechanisms, with a potential contribution of potassium channel activity. However, the available evidence is limited to experimental models, and further in vivo studies are required to determine the physiological relevance and therapeutic potential of this effect.

### 5.5. Antifeedant Activity of Dictamnine

The antifeedant activity of dictamnine has been investigated against stored-product insect pests. Liu et al. [61] reported that dictamnine exhibited feeding-deterrent effects against *Tribolium castaneum* and *Sitophilus zeamais*. The compound showed antifeedant activity against both adult and larval *T. castaneum*, with EC_50_ values of 57.6 and 47.9 ppm, respectively, and against adult *S. zeamais*, with an EC_50_ value of 91.7 ppm. Although dictamnine was less potent than the co-occurring fraxinellone and the commercial feeding deterrent azadirachtin, its activity was comparable to that of toosendanin. The authors suggested that the observed reduction in insect growth was primarily associated with behavioral effects, particularly feeding deterrence and consequent starvation, rather than post-ingestive toxicity, as food utilization was largely unaffected except at higher concentrations.

## 6. Pharmacokinetics, Metabolism, and Toxicity of Dictamnine

### 6.1. Pharmacokinetics and Metabolism of Dictamnine

The pharmacokinetic properties of bioactive compounds are important determinants of their efficacy and safety. Available studies indicate that dictamnine undergoes rapid distribution, has limited oral bioavailability, and is extensively metabolized in the liver, with pronounced interspecies differences in its biotransformation [141,142].

Following intravenous administration in rats, dictamnine rapidly reached high plasma concentrations, whereas oral administration resulted in rapid absorption but limited bioavailability, suggesting substantial first-pass metabolism. The compound was distributed to several organs, with the highest concentrations detected in the liver, kidneys, and lungs. Lower levels in the brain suggested limited penetration across the blood–brain barrier. Only a small proportion of dictamnine was excreted unchanged, indicating that metabolic transformation represents a major route of elimination [141].

Subsequent studies using liver microsomes from mice, rats, dogs, monkeys, and humans demonstrated that dictamnine undergoes CYP450-mediated biotransformation [142]. A key metabolic reaction involves oxidative bioactivation of the 2,3-olefinic moiety of the furan ring, resulting in the formation of the 2,3-epoxide metabolite M7. Among the CYP isoforms investigated, CYP3A4 was identified as the predominant enzyme involved in M7 formation, whereas CYP1A2 and CYP2A6 may also contribute to this metabolic pathway [142]. M7 was identified as a major metabolite of dictamnine, and subsequent epoxide-ring opening was proposed to contribute to the formation of additional metabolites [142]. More generally, in furan-containing compounds, epoxide intermediates are electrophilic and may react with cellular nucleophiles, including glutathione and protein thiol groups; the balance between bioactivation and detoxification pathways may influence the resulting toxicity [143].

Marked interspecies differences were observed in dictamnine metabolism across the species investigated, with differences in the extent and profiles of biotransformation between rodent and human liver microsomes [142]. These findings highlight the limitations of extrapolating animal pharmacokinetic and toxicological data to humans and suggest that species-specific CYP activity should be considered when evaluating the translational potential of dictamnine.

Importantly, the same metabolic pathways involved in dictamnine elimination may also contribute to its toxicity. CYP450-dependent oxidation of the furan moiety leads to the formation of reactive epoxide metabolites that can undergo conjugation with GSH and potentially react with other cellular nucleophiles. Such metabolic activation may compromise hepatic GSH homeostasis and promote oxidative stress, providing a plausible mechanistic link between CYP3A-mediated bioactivation of dictamnine and hepatocellular injury. Consistent with this proposed mechanism, oxidative stress, changes in mitochondrial membrane potential, early apoptotic responses, and disturbances in lipid metabolism have been experimentally associated with dictamnine exposure [144].

### 6.2. Toxicity of Dictamnine

Despite its diverse biological activities, the potential toxicity of dictamnine warrants careful consideration in the context of its further development. The main safety concerns identified to date include hepatotoxicity, phototoxicity, and mutagenicity. The experimentally reported toxicological profile of dictamnine is summarized in Table 3.

#### 6.2.1. Hepatotoxicity

Available evidence indicates that dictamnine-induced hepatotoxicity is strongly associated with metabolic bioactivation rather than with direct toxicity of the parent compound. Feng et al. [150] demonstrated the formation of a reactive epoxide-derived metabolite of dictamnine by identifying an N-acetylcysteine conjugate in microsomal incubations, and in the bile and urine of rats. These findings support the formation of an electrophilic metabolite capable of reacting with cellular nucleophiles. The involvement of CYP3A4 in this metabolic activation was further supported by Li et al. [145], who demonstrated that dictamnine-induced cytotoxicity was associated with CYP3A4 expression and activity, whereas CYP3A4 inhibition attenuated and CYP3A4 induction exacerbated dictamnine-induced liver injury (Table 3).

Subsequent in vivo studies by Shi et al. [146] provided more direct evidence linking CYP3A-dependent metabolic activation to dictamnine-induced hepatotoxicity. Reactive epoxide-derived metabolites were detected as GSH conjugates in liver tissue and as mercapturic acid conjugates in urine, whereas inhibition of CYP3A activity significantly reduced both reactive metabolite formation and liver injury. Furthermore, depletion of hepatic GSH enhanced dictamnine-induced hepatotoxicity, supporting the protective role of GSH in the detoxification of reactive metabolites. The characteristic hepatocellular injury and centrilobular necrosis observed in mice further support the involvement of reactive metabolites in the mechanism of cellular injury [146] (Table 3).

Complementary evidence from integrated transcriptomic and metabolomic analyses was provided by Li et al. [147], who identified changes in pathways related to cytochrome P450-mediated xenobiotic metabolism, glutathione metabolism, ABC transporters, bile secretion, and lipid metabolism following high-dose dictamnine exposure in mice. The authors also observed differential expression of CYP2E1, UGT1A9, and Idh2, suggesting that, in addition to CYP3A-dependent bioactivation, alterations in xenobiotic metabolism and mitochondrial redox homeostasis may contribute to dictamnine-induced hepatotoxicity. Dictamnine-induced hepatotoxicity has also been associated with oxidative stress and early apoptotic responses. Tu et al. [144] reported increased oxidative stress, an elevated Bax/Bcl-2 ratio, and impaired antioxidant defenses, including reduced levels of superoxide dismutase, catalase, and glutathione peroxidase 1. Changes in mitochondrial membrane potential and reactive oxygen species levels were also linked to dictamnine-induced cytotoxicity and apoptosis [144]. More recently, Luo et al. [151] provided further mechanistic insight by demonstrating that CYP3A-dependent bioactivation of dictamnine promoted covalent modification in hepatic proteins by endogenous aldehydes, whereas CYP3A inhibition or aldehyde trapping attenuated protein adduct formation and liver injury. These findings suggest that aldehyde-mediated protein modification may represent an additional downstream mechanism contributing to dictamnine-induced hepatotoxicity. Consistent with the role of metabolic activation, Chang et al. [152] and Lin et al. [153] showed that *Schisandra*-derived lignans attenuated dictamnine-induced hepatotoxicity by inhibiting dictamnine metabolic activation, thereby reducing reactive metabolite formation, GSH depletion, and protein adduct formation.

Evidence obtained using *Cortex Dictamni* preparations further supports the hepatotoxic potential of the plant material, although such findings cannot automatically be attributed to dictamnine alone. Fan et al. [154] investigated the subchronic toxicity of *Cortex Dictamni* extracts in mice and explored their potential hepatotoxic mechanisms in vitro. The administration of the extracts resulted in hepatic injury, with toxic effects associated with oxidative stress, alterations in mitochondrial membrane potential, increased membrane permeability, and induction of apoptosis. Similarly, Gao et al. [155] investigated the hepatotoxicity of *Cortex Dictamni* using network pharmacology, molecular docking, and pharmacological experiments. CYP1A2 and GSR were identified among the potential targets associated with toxicity, while in vitro and in vivo experiments indicated that *Cortex Dictamni* reduced glutathione peroxidase activity and increased oxidative stress, accompanied by changes in CYP1A2 expression. Sun et al. [156] further demonstrated that administration of the whole *Cortex Dictamni* extract induced mitochondrial oxidative stress, increased mitochondrial reactive oxygen species production, altered hepatocyte energy metabolism, and promoted hepatocyte apoptosis. Although dictamnine, obacunone, and fraxinellone were identified among the major constituents of the extract, the mechanistic experiments were performed using the whole extract; therefore, these effects cannot be specifically attributed to dictamnine. Further evidence was provided by Li et al. [157], who reported that the petroleum ether fraction of *Cortex Dictamni* exhibited the strongest hepatotoxicity and was enriched in furan-containing constituents, particularly furanoterpenoids. The observed hepatic injury was associated with disturbances in retinol, histidine, glutathione, and drug metabolism, together with oxidative stress and mitochondrial dysfunction. These results suggest that multiple furan-containing constituents may contribute to the hepatotoxicity of *Cortex Dictamni*, rather than dictamnine being solely responsible for the observed effects. More recently, Liu et al. [158] applied an integrated strategy combining serum pharmacochemistry, metabolomics, network toxicology, molecular docking, molecular dynamics simulations, and in vivo and in vitro experiments to investigate the hepatotoxicity of *Cortex Dictamni.* A total of 34 serum-exposed constituents were identified, among which dictamnine and fraxinellone were proposed as the principal candidate toxic components. *Cortex Dictamni* administration induced hepatic injury and was associated with disturbances in bile acid, lipid, and sphingolipid metabolism. Further experiments demonstrated that dictamnine and fraxinellone promoted hepatocyte apoptosis through modulation of the PI3K/AKT signaling pathway. These findings provide additional evidence that dictamnine may represent an important contributor to the hepatotoxicity of *Cortex Dictamni*, although synergistic or antagonistic interactions among multiple constituents cannot be excluded.

It is also important to note that the hepatic effects of *Dictamnus dasycarpus* may vary depending on the preparation and experimental conditions. Li et al. [159] reported that an aqueous extract of *D. dasycarpus* alleviated α-naphthylisothiocyanate-induced cholestatic liver injury in mice. This effect was associated with restoration of bile acid homeostasis through the FXR/SHP pathway, modulation of the gut microbiota, and attenuation of hepatic fibrosis. However, because this study investigated a complex aqueous extract rather than purified dictamnine, the hepatoprotective effects cannot be directly attributed to dictamnine.

In summary, the available evidence indicates that dictamnine may contribute to the hepatotoxicity of *Cortex Dictamni*, with metabolic bioactivation, oxidative stress, mitochondrial dysfunction, and apoptosis representing important mechanisms underlying this process. Nevertheless, the hepatotoxicity of this herbal drug is likely attributable to the combined effects of multiple constituents, particularly compounds containing a furan ring.

#### 6.2.2. Phototoxicity

Phototoxicity represents another important safety concern. Schempp et al. [160] described two cases of bullous phototoxic contact dermatitis following exposure to *Dictamnus albus* L., in which dictamnine was identified among the plant constituents implicated in the phototoxic response. The phototoxic potential of dictamnine itself was subsequently investigated by Schempp et al. [148] in human Jurkat T lymphocytes and HaCaT keratinocytes. Dictamnine isolated from the roots of *D. albus* exhibited concentration- and light-dependent phototoxicity following irradiation with simulated sunlight. However, its phototoxic activity was lower than that of the structurally related furocoumarins 5-methoxypsoralen and 8-methoxypsoralen. Despite its lower potency, the authors suggested that dictamnine may contribute to phytophotodermatitis because of its abundance in plants of the Rutaceae family.

#### 6.2.3. Mutagenicity

Potential mutagenicity has also been reported. Mizuta and Kanamori [149] demonstrated that dictamnine induced dose-dependent mutagenic activity in *Salmonella typhimurium* TA98 and TA100 in the presence of metabolic activation, with weaker activity also observed in TA100 in its absence. These findings are consistent with the possibility that reactive metabolites generated during dictamnine biotransformation contribute to its mutagenic effects. This hypothesis was further supported by Häfele and Schimmer [89], who showed that dictamnine and other furoquinoline alkaloids were strongly mutagenic in *S. typhimurium* TA98 and TA100 following activation by rat liver microsomal preparations. The mutagenic activity was reduced by several inhibitors of cytochrome P450 and related monooxygenase systems, suggesting that enzymatic biotransformation, particularly via cytochrome P450/P448-dependent pathways, is involved in the formation of mutagenic metabolites. Moreover, the preferential activity of these alkaloids in TA98 and TA100, together with their relatively weak or absent activity in the corresponding non-R-factor strains TA1538 and TA1535, suggested a predominant frameshift mutagenic mechanism [106]. However, the relevance of these bacterial findings to mammalian systems and human health remains unclear and requires further investigation.

Given the potential toxicity of dictamnine, strategies aimed at improving its therapeutic index may include targeted drug-delivery systems and structural modification. Lin et al. [122] demonstrated that PLGA-based nanoparticles enhanced local delivery of dictamnine and improved its anti-inflammatory efficacy in an experimental dermatitis model. Although their effect on systemic toxicity was not directly established, localized delivery may represent a potential approach to reducing unnecessary systemic exposure. Another strategy is the development of dictamnine analogues with modified structural features that reduce metabolic bioactivation while retaining desirable pharmacological properties. Such approaches will require systematic structure–activity relationship studies and comprehensive toxicological evaluation.

## 7. Biological Activity of Other Quinoline Alkaloids from Dictamnus

Beyond dictamnine, the genus *Dictamnus* is a rich source of structurally diverse quinoline alkaloids, with furoquinoline derivatives representing one of the most characteristic groups. Among the compounds identified to date are skimmianine, γ-fagarine, kokusaginine, robustine, isomaculosidine, and several structurally related alkaloids. These alkaloids exhibit diverse biological activities, including anti-inflammatory, cytotoxic, antimicrobial, enzyme-modulating, antiplatelet, and potentially neuroprotective effects. However, compared with dictamnine, most of these compounds have received considerably less attention in studies of their biological and pharmacological properties.

### 7.1. Anti-Inflammatory and Neuroprotective Activity

Anti-inflammatory activity is among the best-documented biological properties of quinoline alkaloids from *Dictamnus*. Yoon et al. [95] isolated one new quinoline alkaloid glycoside and nine known alkaloids from the methanolic extract of the root bark of *D. dasycarpus*. All isolated compounds inhibited LPS-induced nitric oxide (NO) production in BV2 microglial cells, although their potency varied considerably. The strongest inhibitory effects were observed for 8-methoxy-*N*-methylflindersine and skimmianine, with IC_50_ values of 0.4 and 7.0 µM, respectively. Other alkaloids, including preskimmianine, γ-fagarine, haplopine, dictangustine-A, iso-γ-fagarine, and isomaculosidine, also showed inhibitory activity but were less potent [95].

Further studies indicate that *Dictamnus* furoquinoline alkaloids may also modulate inflammatory responses in neutrophils. Sun et al. [10] isolated two dimeric furoquinoline alkaloids, anstifolines A and B, from the root bark of *D. angustifolius*. Both compounds inhibited superoxide anion generation and elastase release in FMLP/CB-stimulated human neutrophils. The dimeric alkaloids showed stronger activity than the corresponding monomeric compounds tested in study, including dictamnine and robustine, suggesting that structural features associated with dimerization may influence the anti-inflammatory activity of furoquinoline alkaloids. Anstifoline A inhibited superoxide anion generation and elastase release with IC_50_ values of 13.0 and 19.6 µM, respectively, whereas the corresponding values for anstifoline B were 17.4 and 12.1 µM. These findings broaden the potential anti-inflammatory mechanisms of *Dictamnus* alkaloids beyond inhibition of NO production [10].

Anti-inflammatory activity has also been reported among compounds obtained from *Cortex Dictamni*. Yang et al. [43] evaluated 31 isolated compounds for their ability to inhibit LPS-induced NO production in RAW 264.7 macrophages. Several alkaloids and phenolic constituents showed significant activity at 40 µM, including haplopine, whereas dictamnine did not significantly inhibit NO production under these experimental conditions. These findings indicate that anti-inflammatory activity is not a uniform property of *Dictamnus* alkaloids and may depend strongly on the individual structure, cellular model, and experimental conditions.

Further evidence was provided by Gao et al. [5], who isolated 24 alkaloids from *D. dasycarpus*, including six previously undescribed compounds. Preskimmianine, dasycarine E, and 4-methoxy-1-methylquinolin-2-one strongly inhibited NO production in LPS-stimulated BV2 cells, with IC_50_ values below 5 µM, whereas nine additional compounds showed moderate activity, with IC_50_ values ranging from 7.8 to 28.4 µM. Moreover, preskimmianine reduced the production of pro-inflammatory mediators, including TNF-α and IL-6, and inhibited activation of the NF-κB signaling pathway. These findings indicate that quinoline alkaloids present in *D. dasycarpus* constitute an important group of compounds with potential anti-inflammatory activity and can modulate multiple inflammatory mediators; however, the evidence remains predominantly based on cellular models.

### 7.2. Cytotoxic and Anticancer Activity

Several *Dictamnus* alkaloids have been shown to exhibit cytotoxic or antiproliferative activity. Kim et al. [55] demonstrated cytotoxic activity of the furoquinoline alkaloids dictamnine and preskimmianine, together with the degraded limonoid fraxinellone, isolated from the roots of *D. albus*, against the L1210 cell line. Among these compounds, preskimmianine exhibited the strongest cytotoxic activity, with an IC_50_ value of 3.125 μg/mL (10.3 μM).

Similarly, Sun et al. [11] reported weak cytotoxic activity of six alkaloids isolated from *D. angustifolius*—5-methoxylrobustine, dictamnine, robustine, isopteleine, γ-fagarine, and skimmianine—against MCF-7 breast cancer cells. Among the tested compounds, robustine exhibited the strongest antiproliferative activity.

Moreover, anstifolines A and B, two dimeric furoquinoline alkaloids isolated from the root bark of *Dictamnus angustifolius*, exhibited moderate cytotoxic activity against the human lung cancer cell lines A549 and NCI-H460 in the MTT assay [10]. Anstifoline A showed IC_50_ values of 13.29 and 14.31 μM, whereas anstifoline B displayed IC_50_ values of 10.64 and 13.18 μM against A549 and NCI-H460 cells, respectively. Both dimeric alkaloids were more cytotoxic than the monomeric furoquinoline alkaloids dictamnine and robustine tested in the same study. This finding suggests that structural modification through dimerization may be associated with enhanced cytotoxic activity, although the contribution of the specific 8–7′ coupling pattern cannot be established solely from these comparative data. Nevertheless, the cytotoxicity of anstifolines A and B was substantially weaker than that of the reference compound voreloxin, which showed an IC_50_ value of 0.39 μM against A549 cells, indicating moderate in vitro potency [10].

Chen et al. [94] showed that kokusaginine isolated from *D. dasycarpus* inhibited the proliferation of human breast cancer cells, including multidrug-resistant (MDR) cell lines. Its activity was associated with induction of apoptosis, inhibition of P-glycoprotein (P-gp) expression and activity, and disruption of tubulin polymerization. The latter mechanism may interfere with microtubule dynamics and mitotic progression, providing a possible explanation for the observed antiproliferative effect in MDR cancer cells.

### 7.3. Antimicrobial Activity

Tian et al. [6] demonstrated antimicrobial activity among quinoline alkaloids isolated from the root bark of *D. dasycarpus*. Robustine and γ-fagarine also showed moderate activity against *B. subtilis* and *C. albicans*, with MIC values ranging from 32 to 64 µg/mL, whereas most of the other tested alkaloids were inactive at concentrations up to 128 µg/mL. These findings further indicate substantial structure-dependent differences in the antimicrobial activity of *Dictamnus* alkaloids [6].

### 7.4. Enzyme-Modulating and Antiplatelet Activity

Some furoquinoline alkaloids also modulate the activity of enzymes involved in vascular signaling. Nam et al. [92] demonstrated that haplopine, robustine, dictamnine, and γ-fagarine isolated from *D. albus* inhibited human phosphodiesterase type 5 (PDE5A) in vitro. γ-Fagarine was the most potent inhibitor, and structure–activity analysis indicated that the position of the methoxy group influenced PDE5A inhibitory activity. Because PDE5 regulates intracellular cGMP levels, inhibition of this enzyme may promote smooth muscle relaxation; however, the reported findings are limited to in vitro enzyme assays and do not establish vasorelaxant efficacy in vivo.

Antiplatelet effects have also been reported for alkaloids from *D. angustifolius*. Sun et al. [7] found that isopteleine, robustine, γ-fagarine, isodictamnine, skimmianine, and 8-hydroxy-9-methylfuro [2,3-*b*]quinolin-4(9H)-one inhibited ADP-induced platelet aggregation in vitro. Although some compounds showed activity comparable to or greater than that of aspirin at the tested concentration, the relatively high concentrations required and the lack of in vivo evidence limit conclusions regarding their therapeutic relevance.

### 7.5. Safety Considerations

As with dictamnine, the biological activity of other *Dictamnus* alkaloids should be considered alongside the limited available evidence regarding their potential toxicity. Shi et al. [161] reported that isomaculosidine activated the NLRP3 inflammasome in response to ATP or nigericin, an effect associated with increased mitochondrial reactive oxygen species (ROS) production and proposed as a potential mechanism contributing to idiosyncratic liver injury. Potential genotoxicity has also been reported for *Dictamnus* alkaloids. Mizuta and Kanamori [149] demonstrated mutagenic activity of γ-fagarine and dictamnine in *Salmonella typhimurium*, while Häfele and Schimmer [106] showed that the mutagenicity of furoquinoline alkaloids is strongly influenced by metabolic activation. These findings further support the need to consider the toxicological potential of individual *Dictamnus* alkaloids alongside their biological activities. In addition, studies of whole *Cortex Dictamni* preparations have demonstrated hepatotoxic effects associated with oxidative stress and mitochondrial dysfunction; however, these findings cannot be directly attributed to individual alkaloids, including isomaculosidine or dictamnine [156]. This distinction is important when interpreting the safety of *Dictamnus* preparations, as the observed toxic effects may result from the combined action of multiple constituents, their metabolites, or interactions among them. Therefore, although individual alkaloids have demonstrated specific biological and potentially toxic effects, the contribution of each constituent to the overall toxicity of *Dictamnus* preparations remains to be established.

## 8. Conclusions

**Figure 4:**
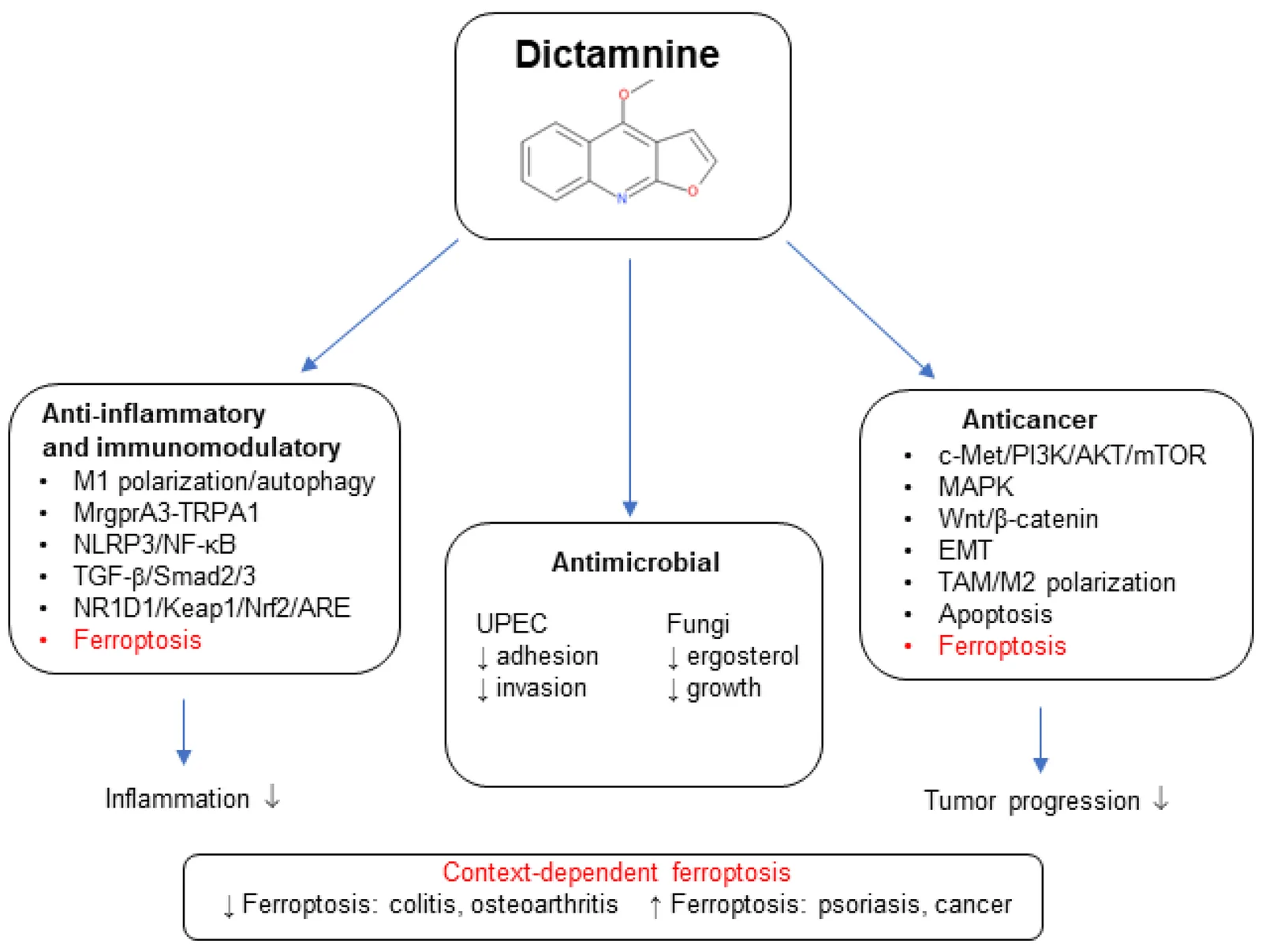
Major biological activities of dictamnine and proposed mechanisms of action.

Quinoline alkaloids constitute an important and structurally diverse group of secondary metabolites characteristic of the genus *Dictamnus*. Among these compounds, particular attention has been devoted to dictamnine, which is currently one of the best-studied furoquinoline alkaloids of this genus. As shown in Figure 4, dictamnine exhibits a broad range of biological activities, reflecting its pleiotropic effects on numerous molecular pathways and cellular processes. These activities include anti-inflammatory, anticancer, antimicrobial, and antifeedant effects, as well as effects on the vascular system. The biological activity of dictamnine has been linked to several cellular and molecular processes such as inflammation-related signaling, apoptosis, autophagy, ferroptosis, epithelial–mesenchymal transition, and the tumor microenvironment.

Other quinoline alkaloids from the genus *Dictamnus*, particularly furoquinoline derivatives such as skimmianine, γ-fagarine, kokusaginine, robustine, and isomaculosidine, also exhibit a diverse range of biological properties, including anti-inflammatory, cytotoxic, antimicrobial, and neuroprotective effects, as well as the ability to modulate enzyme activity and inhibit platelet aggregation. However, these compounds have been investigated considerably less extensively, and their biological activities and mechanisms of action remain less well characterized than those of dictamnine.

The pharmacological potential of quinoline alkaloids, particularly furoquinoline derivatives, from the genus *Dictamnus* should also be considered in the context of their safety profile. Evidence suggesting their potential metabolic activation, hepatotoxicity, phototoxicity, and genotoxicity highlights the need for a thorough assessment of their therapeutic applicability. The findings also emphasize the importance of considering dose, exposure duration, and chemical structure when assessing the safety and biological effects of these compounds. Further studies are needed to determine whether their potential therapeutic effects can be achieved within a sufficiently safe therapeutic window and to better characterize their pharmacokinetic properties, metabolic pathways, and short- and long-term toxicity.

Overall, the available evidence remains predominantly preclinical, and further studies are needed to determine the clinical relevance of the reported biological activities.

## 9. Future Perspectives and Research Directions

Despite the growing body of evidence demonstrating the biological activities of quinoline alkaloids occurring in the genus *Dictamnus*, particularly dictamnine, their pharmacological potential has not yet been fully elucidated. Future research should focus on several key areas, including structure–activity relationships, ADME and pharmacokinetic properties, active metabolites, in vivo validation, safety, potential interactions, and biosynthesis.

A key issue that remains to be addressed is the elucidation of the molecular mechanisms underlying the biological activities of alkaloids occurring in the genus *Dictamnus*. Although mechanisms of action have been proposed for selected compounds, these studies cover a relatively limited number of alkaloids. Integrated molecular, cellular, and biochemical approaches could provide a more comprehensive understanding of the pathways underlying their biological effects. Comparative studies of structurally related furoquinoline alkaloids under standardized experimental conditions would also be valuable for elucidating structure–activity relationships and determining how the core scaffold and the type and position of substituents influence biological activity.

The ADME and pharmacokinetic properties of dictamnine and other furoquinoline alkaloids remain insufficiently characterized. Further studies should address their absorption, distribution, metabolism, excretion, bioavailability, and tissue exposure, with particular attention to metabolic transformations and the identification of biologically active metabolites. Such investigations are essential for determining whether the observed biological effects can be attributed solely to the parent compounds or also to their metabolites.

Further in vivo studies are needed to validate the biological activities reported for dictamnine and other *Dictamnus* alkaloids. In particular, additional investigation is warranted to establish dose–response relationships, the persistence of the observed effects, and their reproducibility in independent models. Equally important is a systematic assessment of safety, including potential toxicity and phototoxicity, and the selectivity of biological effects.

Potential interactions represent another important research gap. Future studies should examine interactions between dictamnine and other *Dictamnus* alkaloids, and with drugs used in clinical practice. The interactions among constituents of *Cortex Dictamni* also warrant investigation, as they may influence the overall biological activity and safety of the crude drug.

Finally, the proposed biosynthetic pathways and the chemotaxonomic significance of quinoline alkaloids in the genus *Dictamnus* require further experimental validation. The integration of metabolomics, transcriptomics, enzymatic studies, and genomic approaches could facilitate the identification of genes and enzymes involved in alkaloid biosynthesis and provide further insight into the structural diversity of these compounds.

Taken together, these research directions should provide a more comprehensive understanding of the biological activities, pharmacological properties, safety, and biosynthetic significance of quinoline alkaloids in the genus *Dictamnus*.

## Figures and Tables

**Table 1:** Representative structures of major quinoline alkaloids illustrating their structural diversity. Structures I–XXV represent selected quinoline alkaloids identified in *Dictamnus* (Figure 1 and Figure 2).

No.	Structure	Compound	R_1_	R_2_	R_3_	R_4_	Plant Taxon	Ref.
1	I	Dictamnine	H	H	H	H	*D. dasycarpus*	[4,61,86,87,88]
							*D. angustifolius*	[3,89]
							*D. albus*	[55,90]
							*D. caucasicus*	[91]
2	I	5-Hydroxydictamnine	OH	H	H	H	*D. angustifolius*	[9]
3	I	Robustine	H	H	H	OH	*D. dasycarpus*	[5]
							*D. angustifolius*	[7]
							*D. albus*	[92]
							*D. caucasicus*	[91]
4	I	5-Methoxydictamnine	OCH_3_	H	H	H	*D. angustifolius*	[8]
5	I	Evolitrine	H	H	OCH_3_	H	*D. albus*	[90]
6	I	γ-Fagarine	H	H	H	OCH_3_	*D. dasycarpus*	[4,86,93]
							*D. angustifolius*	[3,7,8,89]
							*D. albus*	[92,90]
							*D. caucasicus*	[91]
7	I	Kokusaginine	H	OCH_3_	OCH_3_	H	*D. dasycarpus*	[94]
8	I	Maculosidine	H	OCH_3_	H	OCH_3_	*D. albus*	[90]
9	I	Skimmianine	H	H	OCH_3_	OCH_3_	*D. dasycarpus*	[95]
							*D. angustifolius*	[3,7]
							*D. albus*	[90]
							*D. caucasicus*	[91]
10	I	5-Methoxyrobustine	OCH_3_	H	H	OCH_3_	*D. angustifolius*	[11]
11	I	5-Hydroxy-4,8-dimethoxy-furoquinoline	OH	H	H	OCH_3_	*D. angustifolius*	[8]
12	I	Haplopine	H	H	OH	OCH_3_	*D. dasycarpus*	[5,96]
							*D. angustifolius*	[8]
							*D. albus*	[92]
13	I	4,7-Dimethoxy-furo [2,3-*b*]quinolin-8-ol	H	H	OCH_3_	OH	*D. angustifolius*	[9]
14	II	O-Ethylnordictamnine	H	H	H	H	*D. dasycarpus*	[97]
							*D. angustifolius*	[9]
15	II	O-Ethylnor-γ-fagarine	H	H	H	OCH_3_	*D. dasycarpus*	[97]
16	II	O-Ethylnorskimmianine	H	H	OCH_3_	OCH_3_	*D. dasycarpus*	[97]
17	III	Sonminine	-	-	-	-	*D. angustifolius*	[8]
18	IV	Isodictamnine	H	H	H	H	*D. angustifolius*	[3,89]
							*D. albus*	[7,8,98]
							*D. caucasicus*	[91]
19	IV	Dictangustine A	H	OH	H	H	*D. angustifolius*	[3]
20	IV	8-Hydroxy-9-methyl-furo [2,3-*b*]quinolin-4(9H)-one	H	H	H	OH	*D. dasycarpus*	[7,99]
21	IV	Isopteleine	H	OCH_3_	H	H	*D. angustifolius*	[7,8,89]
							*D. caucasicus*	[91]
22	IV	Isoevolitrine	H	H	OCH_3_	H	*D. angustifolius*	[9]
23	IV	Iso-γ-fagarine	H	H	H	OCH_3_	*D. dasycarpus*	[95]
							*D. angustifolius*	[3]
24	IV	Isomaculosidine	H	OCH_3_	H	OCH_3_	*D. dasycarpus*	[95]
							*D. angustifolius*	[3]
							*D. albus*	[90]
							*D. caucasicus*	[91]
25	V	Demethoxyacrophylline	-	-	-	-	*D. dasycarpus*	[6]
26	VI	Myrtopsine	H	H	OH	-	*D. dasycarpus*	[4]
27	VI	7,8-Dimethoxyplatydesmine	OCH_3_	OCH_3_	H	-	*D. dasycarpus*	[93]
							*D. angustifolius*	[9]
28	VI	7,8-Dimethoxymyrtopsine	OCH_3_	OCH_3_	OH	-	*D. dasycarpus*	[4,12,93]
29	VII	1′,2′-Didehydro-7,8-dimethoxyplatydesmine	-	-	-	-	*D. dasycarpus*	[4]
30	VIII	1′-Oxo-isoplatydesmine	-	-	-	-	*D. dasycarpus*	[6]
31	IX	N-methylflindersine	CH_3_	H	-	-	*D. dasycarpus*	[5,6]
							*D. angustifolius*	[3]
32	IX	8-Methoxy-N-methylflindersine	CH_3_	OCH_3_	-	-	*D. dasycarpus*	[6,95]
33	IX	8-Methoxyflindersine	H	OCH_3_	-	-	*D. dasycarpus*	[5,6,12]
34	X	(3R)-(−)-8,9-dimethoxygeibalansine	α–OH	-	-	-	*D. dasycarpus*	[4,12]
35	X	3-Chloro-8,9-dimethoxygeibalansine	Cl	-	-	-	*D. dasycarpus*	[4]
36	XI	5,9-Dimethoxy-2,2-dimethyl-2H-pyrano [2,3-*b*]quinoline	-	-	-	-	*D. dasycarpus*	[4]
37	XII	Glycolone	H	H	-	-	*D. dasycarpus*	[12]
38	XII	Dasycarine	OCH_3_	H	-	-	*D. dasycarpus*	[12,86,100]
							*D. angustifolius*	[9]
39	XII	Preskimmianine	H	OCH_3_	-	-	*D. dasycarpus*	[95]
							*D. angustifolius*	[3,89]
							*D. albus*	[55,90]
40	XIII	Ifflaiamine	-	-	-	-	*D. dasycarpus*	[101,102]
41	XIV	Dubamine	-	-	-	-	*D. angustifolius*	[103]
42	XV	Dasycarine A	-	-	-	-	*D. dasycarpus*	[5]
43	XVI	Dasycarine B	-	-	-	-	*D. dasycarpus*	[5]
44	XVII	Dasycarine C	-	-	-	-	*D. dasycarpus*	[5]
45	XVIII	Dasycarine D	-	-	-	-	*D. dasycarpus*	[5]
46	XIX	Dasycarine E	-	-	-	-	*D. dasycarpus*	[5]
47	XX	Angustine	-	-	-	-	*D. angustifolius*	[104]
48	XXI	2-Hydroxy-4-methoxy-3-(3′-methyl-2′-butenyl)-quinoline	-	-	-	-	*D. dasycarpus*	[5]
49	XXII	4-Methoxy-1-methyl-quinolin-2-one	H	-	-	-	*D. dasycarpus*	[5]
50	XXII	3-Quinolinecarboxylic acid	COOCH_3_	-	-	-	*D. dasycarpus*	[5]
51	XXIII	4-Methoxy-1*H*-quinolin-2-one	H	H	H	-	*D. dasycarpus*	[5]
52	XXIII	Swietenidin B	H	H	OCH_3_	-	*D. dasycarpus*	[5]
53	XXIV	Anstifoline A	-	-	-	-	*D. angustifolius*	[10]
54	XXV	Anstifoline B	-	-	-	-	*D. angustifolius*	[10]

**Table 2:** Biological properties of dictamnine.

Biological Activity	Experimental Model	DIC ^1^ Dose/Concentration	Proposed Mechanisms/Molecular Effects	Key Findings	References
Anti-inflammatory and immunomodulatory activities					
Atopic dermatitis	PMA-differentiated THP-1 macrophages polarized to the M1 phenotype by LPS/IFN-γ stimulation; DNFB-induced AD-like mouse model	50, 100, and 200 µM (in vitro); 5, 10, and 20 mg/kg, p.o. ^2^, once daily, days 2–8 (in vivo)	Inhibition of M1 macrophage polarization and promotion of autophagy	Reduced pro-inflammatory cytokine production, suppressed M1 macrophage polarization, and ameliorated atopic dermatitis-like skin lesions.	[120]
Atopic dermatitis	Activated keratinocytes, macrophages, and basophils; DNCB-induced atopic dermatitis-like mouse model	0–250 µM (in vitro); 20 mM, topical application, days 7, 9, 11, and 13 (in vivo)	Inhibition of keratinocyte, macrophage, and basophil activation	Alleviated dermatitis-like lesions and reduced inflammatory responses.	[121]
Atopic dermatitis	Oxazolone-induced AD-like mouse model	NA ^3^	Enhanced transdermal delivery of dictamnine via PLGA nanocarriers	Improved skin penetration and enhanced anti-inflammatory efficacy, resulting in amelioration of oxazolone-induced atopic dermatitis-like skin lesions.	[122]
Chronic itch	DNFB-induced AD-like mouse model	NA	Inhibition of the MrgprA3-TRPA1 signaling pathway	Significantly alleviated chronic pruritus and skin inflammation.	[123]
Asthma	TGF-β-stimulated human bronchial epithelial (HBE) cells; ovalbumin-induced murine asthma model	50 µM (in vitro); 10 and 20 mg/kg, p.o., once daily, days 11–17 (in vivo)	Suppression of the TGF-β/Smad2/3 signaling pathway	Reduced airway inflammation and remodeling, decreased Th2 cytokine production, MUC5AC expression, and fibrosis, and improved epithelial barrier integrity.	[124]
Allergic rhinitis	LAD2 mast cells; OVA-induced murine allergic rhinitis model	1, 10, 50, 100, and 200 µM (in vitro); 2, 10, and 20 mg/kg, p.o., 3 times/week for 5 weeks (in vivo)	Inhibition of LYN kinase-mediated FcεRI signaling and mast cell activation	Reduced mast cell activation and allergic responses, decreased nasal rubbing and sneezing, and alleviated allergic rhinitis-like symptoms.	[125]
Colitis	RSL3-induced ferroptosis in NCM460 cells; DSS-induced colitis mouse model	10, 20, and 40 mg/kg, p.o., once daily for 10 days (in vivo)	Inhibition of enterocyte ferroptosis via activation of the Nrf2/GPX4 signaling pathway	Alleviated DSS-induced colitis by reducing intestinal inflammation and oxidative stress, and improving intestinal barrier integrity.	[126]
Psoriasis	KGF-stimulated HaCaT keratinocytes; IMQ-induced psoriasis-like dermatitis mouse model	12.5, 25, and 50 µM (in vitro); 5, 10, and 20 mg/kg, p.o. for 9 days (in vivo)	Inhibition of HIF1A signaling, leading to downregulation of GPX4 and SLC7A11, induction of ferroptosis in hyperproliferative keratinocytes; suppression of inflammatory cytokine production	Inhibited keratinocyte hyperproliferation, reduced inflammatory responses, and alleviated psoriasis-like dermatitis.	[127]
Rheumatoid arthritis	TNF-α-induced human fibroblast-like synoviocytes (HFLS-RA); collagen-induced arthritis (CIA) mouse model	6.25 and 12.5 µM (in vitro); 10 and 20 mg/kg, p.o., once daily for 28 days (in vivo)	Activation of the nuclear receptor NR1D1, leading to modulation of the Keap1/Nrf2/ARE antioxidant pathway, reduction in ROS accumulation, and restoration of mitochondrial membrane potential	Inhibited TNF-α-induced HFLS-RA proliferation and synovial hyperplasia in CIA mice via the NR1D1/Keap1/Nrf2/ARE pathway.	[128]
Osteoarthritis	Chondrocytes; OA animal model; human samples	50 and 100 µM (in vitro); 50 and 100 µM, i.a. ^4^, 10 µL every other day for 12 weeks (in vivo)	Inhibition of HIF-1α/HO-1 signaling	Inhibited chondrocyte ferroptosis via the HIF-1α/HO-1 signaling pathway and alleviated OA progression.	[129]
Anticancer activity					
Lung cancer	Human lung cancer cell lines (A549, H1299, PC9, and EBC-1); xenograft mouse model	50, 100, and 200 µM (in vitro); 50 mg/kg, i.p. ^5^ for 15 days (in vivo)	Inhibition of the receptor tyrosine kinase c-Met, leading to suppression of the PI3K/AKT/mTOR and MAPK signaling pathways	Inhibited lung cancer cell proliferation, migration, and invasion and induced apoptosis.	[130]
Colorectal cancer	HCT116 human colorectal cancer cells; xenograft mouse model	10–100 µM (in vitro); 50–100 mg/kg, p.o. (in vivo)	Downregulation of HIF-1α and Slug signaling pathways, leading to inhibition of epithelial-mesenchymal transition (EMT), suppression of migration and invasion	Suppressed cancer cell proliferation, migration, and invasion, inhibited EMT, and promoted apoptosis.	[131]
Colorectal cancer	Human colorectal cancer cell lines (DLD-1, LoVo); human colorectal cancer xenograft mouse model	25, 50, and 100 µM (in vitro); 50 mg/kg, i.p., every 2 days (in vivo)	Induction of ferroptosis; inhibition of M2 macrophage polarization through MAPK signaling	Suppressed tumor growth and remodeled the tumor immune microenvironment.	[132]
Lung adenocarcinoma	Human lung adenocarcinoma A549 cells	100, 300, and 500 µM (in vitro); 100 µM DIC + 6 or 12 µg/mL DHA ^6^ (combination treatment)	Caspase-dependent apoptosis enhanced by dihydroartemisinin	Combination treatment significantly increased apoptosis and inhibited A549 cell proliferation.	[133]
Pancreatic cancer	PANC-1 and BxPC-3 human pancreatic cancer cells; hTERT-HPNE cells; nude mouse xenograft model	20–100 µM (in vitro); 15 mg/kg (i.p., once daily for 4 weeks, in vivo)	Inhibition of PI3K/AKT signaling pathway; suppression of epithelial-mesenchymal transition	Reduced pancreatic cancer cell growth, migration, invasion, and tumor progression.	[134]
Prostate cancer	Human prostate cancer cell lines (DU145, PC-3, and 22Rv1); subcutaneous xenograft mouse model	0–300 µM (in vitro)	Stabilization of DKK1 and inhibition of the Wnt/β-catenin pathway and EMT; suppression of angiogenesis and modulation of the tumor microenvironment	Inhibited prostate cancer cell proliferation, migration, invasion, and tumor growth; reversed EMT, suppressed angiogenesis, and modulated the tumor microenvironment.	[135]
Antimicrobial activity					
Antibacterial	Human urothelial T24 cells infected with the clinical UPEC307 strain	2.5, 5, 10, and 20 µg/mL	Inhibition of UPEC adhesion and invasion through downregulation of UPEC fimbriae-encoding genes and urothelial receptor genes in T24 cells	Inhibited UPEC adhesion to and invasion of urothelial cells without direct antibacterial activity at the tested concentrations.	[136]
Antimicrobial	Human pathogenic microorganisms: *Bacillus subtilis* (ATCC 6633), *Candida albicans* (ATCC 10231), and *Pseudomonas aeruginosa* (ATCC 27853)	8–128 µg/mL	Not investigated	Dictamnine exhibited moderate antimicrobial activity.	[6]
Antifungal	*Cladosporium cucumerinum*	0.6 µg (TLC bioassay)	Not investigated	Dictamnine exhibited strong antifungal activity.	[20]
Antifungal	*Botrytis cinerea* on harvested fruit	20, 50, 200, and 500 μM	Suppression of ergosterol biosynthesis, leading to disruption of fungal cell membrane integrity	Inhibited spore germination and mycelial growth of *B. cinerea* and reduced gray mold lesion development on postharvest fruits in a dose-dependent manner.	[137]
Vasorelaxant activity					
	Isolated rat thoracic aorta rings	10–500 µM	Suppression of Ca^2+^ influx through voltage-dependent and receptor-operated Ca^2+^ channel	Induced concentration-dependent relaxation of isolated rat thoracic aorta.	[138]
Antifeedant activity					
	*T. castaneum* (adults/larvae), *S. zeamais* (adults); feeding-deterrence assays	3–300 ppm	Behavioral feeding deterrence; reduced growth mainly attributed to starvation rather than post-ingestive toxicity	Inhibited feeding, with EC_50_ values of 57.6 ppm (*T. castaneum* adults), 47.9 ppm (*T. castaneum* larvae), and 91.7 ppm (*S. zeamais* adults).	[61]

^1^ DIC, dictamnine; ^2^ p.o., per os (oral administration); ^3^ NA, not accessible, ^4^ i.a., intra-articular injection; ^5^ i.p., intraperitoneal; ^6^ DHA, dihydroartemisinin.

**Table 3:** Experimental evidence of the toxicological effects of dictamnine.

Toxicity Type	Experimental Model	DIC ^1^ Dose/Concentration	Exposure Duration	Observed Cellular/Organ Effects	Toxicological Endpoint	References
Hepatotoxicity	PHH ^2^, HepG2 and L02 cell lines, in vitro	100–800 μM	24–48 h	Concentration-dependent reduction in cell viability; enhanced by CYP3A4 induction with RIF ^3^ and attenuated by CYP3A4 inhibition with KTZ ^4^	Cytotoxicity	[145]
Hepatotoxicity	ICR mice, in vivo; oral administration of DIC	320 mg/kg for 4 days, followed by 640 mg/kg on day 5	5 days	Hepatocellular degeneration and congestion; increased ALT ^5^ and AST ^6^ activities; enhanced by DEX ^7^ and attenuated by KTZ	Hepatocellular injury	[145]
Hepatotoxicity	Mice, in vivo; single intraperitoneal administration of DIC	150 mg/kg	6–12 h	Hydropic swelling and hepatocyte ballooning degeneration around the central vein	Hepatocellular injury	[146]
	Mice, in vivo; single intraperitoneal administration of DIC	150 mg/kg	24 h	Hepatocellular necrosis in the centrilobular region; increased serum ALT	Hepatocellular injury	
	Mice, in vivo; single intraperitoneal administration of DIC	200 mg/kg	24 h	Massive zonal necrosis; increased serum ALT	Hepatocellular injury	
	Mice, in vivo; single intraperitoneal administration of DIC	150 mg/kg	0.25–4 h	Reduced hepatic GSH ^8^ levels, with ~73% depletion at 0.75 h	Hepatic GSH depletion	
	Mice, in vivo; BSO ^9^ pretreatment followed by a single intraperitoneal administration of DIC	100 mg/kg	24 h	Severe zonal necrosis; serum ALT increased 137-fold compared with DIC alone	Hepatocellular injury	
Hepatotoxicity	Male ICR mice, in vivo; single oral administration of DIC	4 or 640 mg/kg	24 h	At 640 mg/kg: hepatocellular degeneration, steatosis, and necrosis; no significant changes at 4 mg/kg	Hepatocellular injury	[147]
Hepatotoxicity	HepaRG cells, in vitro	20, 50, 100 μM	48 h	Dose-dependent inhibition of proliferation; increased ROS and lipid accumulation; altered mitochondrial membrane potential and GSH levels; increased apoptosis at higher concentration	Cytotoxicity	[144]
Hepatotoxicity	Female ICR mice, in vivo; oral administration of DIC	0.6, 3, 15 mg/kg/day	4 weeks	At 15 mg/kg/day hepatocellular vesicular steatosis; increased AST, TBIL ^10^ and DBIL ^11^; increased relative liver weight; changes in markers related to oxidative stress, apoptosis and lipid metabolism	Hepatocellular injury	[144]
Phototoxicity	Human Jurkat T cells and HaCaT keratinocytes; in vitro; solar-simulated radiation	Up to 80 µg/mL	2 h dark incubation, followed by solar-simulated irradiation	Concentration- and light-dependent decrease in ^3^H-thymidine uptake	Inhibition of cell proliferation	[148]
Mutagenicity	*Salmonella typhimurium* TA98 and TA100; Ames test with PCB-induced rat-liver S9 metabolic activation	10–40 µg/ plate	NR ^12^	Dose-dependent increase in His^+^ revertant colonies; ~70 revertants/µg in TA100 and ~50 revertants/µg in TA98	Bacterial gene mutation (frameshift)	[149]
Mutagenicity	*Salmonella typhimurium* TA98, TA100, TA1538 and TA1535; Ames test with Pb-induced rat-liver microsomal metabolic activation	0.5–40 µg/ plate	NR	Increased His^+^ revertant frequency, particularly in TA98 and TA100; mutagenic response increased with microsomal protein concentration and was reduced by several enzyme inhibitors	Bacterial gene mutation (frameshift)	[106]

^1^ DIC, dictamnine; ^2^ PHHs, primary human hepatocytes; ^3^ RIF, rifampicin; ^4^ KTZ, ketoconazole; ^5^ ALT, alanine aminotransferase; ^6^ AST, aspartate aminotransferase; ^7^ DEX, dexamethasone; ^8^ GSH, glutathion; ^9^ BSO, L-buthionine-S,R-sulfoximine; ^10^ TBIL, total bilirubin; ^11^ DBIL, direct bilirubin; ^12^ NR, not reported.

## Data Availability

No new data were created or analyzed in this study. Data sharing is not applicable to this article.

## Abbreviations

The following abbreviations are used in this manuscript:

AD	Atopic dermatitis
AKT	Protein kinase B
ALT	Alanine aminotransferase
AST	Aspartate aminotransferase
BSO	L-buthionine-S,R-sulfoximine
c-Met	Cellular mesenchymal-epithelial transition factor
CYP450	Cytochrome P450
DBIL	Direct bilirubin
DEX	Dexamethasone
DHA	Dihydroartemisinin
DIC	Dictamnine
DKK1	Dickkopf-related protein 1
DNCB	2,4-Dinitrochlorobenzene
DNFB	2,4-Dinitrofluorobenzene
DSS	Dextran sulfate sodium
EMT	Epithelial–mesenchymal transition
ERK	Extracellular signal-regulated kinase
GPX4	Glutathione peroxidase 4
GSH	Glutathion
FXR/SHP	Farnesoid X receptor/small heterodimer partner
HBE	Human bronchial epithelial cells
HGF	Hepatocyte growth factor
HIF-1α	Hypoxia-inducible factor 1-alpha subunit
HIF1A	Gene encoding hypoxia-inducible factor 1 alpha subunit
HO-1	Heme oxygenase-1
hPDE5A	Human phosphodiesterase-5A
IFN-γ	Interferon-γ
IL-1β	Interleukin-1 beta
IL-6	Interleukin-6
IMQ	Imiquimod
KTZ	Ketoconazole
LPS	Lipopolysaccharide
MAPK	Mitogen-activated protein kinase
MDR	Multidrug resistance
MrgprA3	Mas-related G protein-coupled receptor A3
mTOR	Mechanistic target of rapamycin
MUC5AC	Mucin 5AC
NF-κB	Nuclear factor kappa B
NLRP3	NOD-, LRR- and pyrin domain-containing protein 3
Nrf2	Nuclear factor erythroid 2-related factor 2
NR1D1	Nuclear receptor subfamily 1 group D member 1, also known as Rev-erbα
PHHs	Primary human hepatocytes
PI3K	Phosphoinositide 3-kinase
PLGA	Poly(lactic-co-glycolic acid)
PMA	Phorbol 12-myristate 13-acetate
RIF	Rifampicin
ROS	Reactive oxygen species
Slug (SNAI2)	Snail family transcriptional repressor 2
TAM	Tumor-associated macrophages
TGF-β	Transforming growth factor beta
TBIL	Total bilirubin
TNF-α	Tumor necrosis factor alpha
TRPA1	Transient receptor potential channel A1
UPEC	Uropathogenic *Escherichia coli*
OA	Osteoarthritis
